# Cannabinoid Receptor 1 Inhibition in Chronic Kidney Disease: A New Therapeutic Toolbox

**DOI:** 10.3389/fendo.2021.720734

**Published:** 2021-07-07

**Authors:** Myriam Dao, Helene François

**Affiliations:** ^1^ INSERM UMR_S 1155, Hôpital Tenon, Sorbonne Université, Paris, France; ^2^ AP-HP, Néphrologie et Transplantation Rénale Adulte, Hôpital Necker Enfants Malades, Paris, France; ^3^ AP-HP, Soins Intensifs Néphrologiques et Rein Aigu (SINRA), Hôpital Tenon, Sorbonne Université, Paris, France

**Keywords:** cannabinoid, cannabinoid receptor type 1, chronic kidney disease, endocannabinoids, renal fibrosis

## Abstract

Chronic kidney disease (CKD) concerns millions of individuals worldwide, with few therapeutic strategies available to date. Recent evidence suggests that the endocannabinoid system (ECS) could be a new therapeutic target to prevent CKD. ECS combines receptors, cannabinoid receptor type 1 (CB1R) and type 2 (CB2R), and ligands. The most prominent receptor within the kidney is CB1R, its endogenous local ligands being anandamide and 2-arachidonoylglycerol. Therefore, the present review focuses on the therapeutic potential of CB1R and not CB2R. In the normal kidney, CB1R is expressed in many cell types, especially in the vasculature where it contributes to the regulation of renal hemodynamics. CB1R could also participate to water and sodium balance and to blood pressure regulation but its precise role remains to decipher. CB1R promotes renal fibrosis in both metabolic and non-metabolic nephropathies. In metabolic syndrome, obesity and diabetes, CB1R inhibition not only improves metabolic parameters, but also exerts a direct role in preventing renal fibrosis. In non-metabolic nephropathies, its inhibition reduces the development of renal fibrosis. There is a growing interest of the industry to develop new CB1R antagonists without central nervous side-effects. Experimental data on renal fibrosis are encouraging and some molecules are currently under early-stage clinical phases (phases I and IIa studies). In the present review, we will first describe the role of the endocannabinoid receptors, especially CB1R, in renal physiology. We will next explore the role of endocannabinoid receptors in both metabolic and non-metabolic CKD and renal fibrosis. Finally, we will discuss the therapeutic potential of CB1R inhibition using the new pharmacological approaches. Overall, the new pharmacological blockers of CB1R could provide an additional therapeutic toolbox in the management of CKD and renal fibrosis from both metabolic and non-metabolic origin.

## Introduction

Chronic kidney disease (CKD) is an important problem worldwide and remains a burden for public health (1). CKD corresponds to the replacement of renal functional tissue by extracellular matrix proteins that progressively and irreversibly alter renal function, i.e renal fibrogenesis. Although only 1% of people with CKD reach end-stage renal disease (ESRD), it remains the most expensive of chronic diseases and significantly reduces lifespan (1). To date, only the renin angiotensin system (RAS) blockade using either angiotensin converting enzyme inhibitors or angiotensin II receptor blockers has been shown to be effective in slowing the progression of CKD (2). Recently, new therapeutic classes have improved the management of diabetic nephropathy, which is the main cause of CKD and ESRD worldwide (3), particularly sodium glucose co-transporter 2 (SGLT2) inhibitors (4–8) and glucagon-like peptide-1 receptor (GLP-1R) agonists (9, 10). SGLT2 inhibitors can prevent CKD progression, ESRD and death from renal or cardiovascular causes and are currently approved for use in adults with type 2 diabetes by the U.S. Food and Drug Administration. Interestingly, SGLT2 inhibitors could be efficient regardless of the presence or absence of diabetes (4–8). There is therefore a critical need for new therapeutics in CKD especially in non-metabolic nephropathies. A growing body of evidence suggests that the endocannabinoid system (ECS) could be a new therapeutic target to prevent CKD (11–18). The ECS includes two receptors, the cannabinoid receptor type 1 (CB1R) and the cannabinoid receptor type 2 (CB2R), and about sixty endogenous ligands. The foremost exogenous ligand is Δ9-tetrahydrocannabinol (Δ9-THC). CB1R was the first endocannabinoid receptor to be identified within the brain (19). CB1R belongs to the 7-transmembrane regions receptor family and is a G-protein coupled receptor (19, 20). CB1R is best known to be involved in the regulation of addictive behavior, emotional behavior, analgesia, and memory (21, 22). CB1R is also involved in metabolic pathways in peripheral tissues (11, 23). A second G-protein coupled receptor, CB2R, was later discovered. CB2R is mainly expressed in the immune system and it contributes to its regulation (20). The best-known endogenous ligands are N -arachidonoylethanolamine (anandamide, AEA) and 2-arachidonoylglycerol (2-AG). Most endogenous ligands are ecosanoïds which derive from phospholipids of cellular membranes (24, 25). Whereas CB1R expression is low in normal kidneys (12, 26–28), we previously found that its expression increased in metabolic CKD but also in non-metabolic CKD such as IgA nephropathy, acute interstitial nephritis, and chronic allograft nephropathy (12, 28). Recent studies have demonstrated that CB1R is involved in CKD during diabetes and/or obesity (13, 15–18) by its role on metabolism but also through a direct action on podocytes and tubules. Activation of CB1R enhances oxidative stress, inflammation, and fibrosis within the kidney, whereas CB2R exerts opposite effects. However, CB2R agonists do not provide any additive effect to CB1R inhibition (11, 12). We will therefore mainly focus on CB1R inhibition as a novel target in CKD. The first human clinical trials exploring the therapeutic potential of the ECS were designed to investigate the effect of CB1R inhibition in obesity and metabolic syndrome. Rimonabant, a CB1R inverse agonist which belongs to the first generation of CB1R antagonists, was the first approved for clinical use (29–36). However, due to central nervous side effects, mainly depression, rimonabant was withdrawn from the market in 2007. Since then, compounds with minimal blood brain barrier (BBB) penetration, such as AM645 or JD5037, have been developed (12, 16, 18, 37–40). Recently, third generation CB1R antagonists, which are peripherally restricted dual-target CB1R antagonists, have been designed to improve therapeutic efficacy by targeting a second pathway involved in the pathological conditions (37, 41). More and more compounds are under development to both prevent BBB penetration and enhance peripheral CB1R inhibition efficacy, including β-Arrestin-2 biased peripheral CB1R antagonist (42) and CB1R blocking antibodies (NCT03261739; NCT03900325 clinical studies ongoing). In the present review, we will first describe the role of the endocannabinoid receptors, especially CB1R, in renal physiology. We will next explore the role of endocannabinoid receptors in both metabolic and non-metabolic CKD and renal fibrosis. Finally, we will discuss the therapeutic potential of CB1R inhibition using the newest pharmacological approaches.

## The Role of the Endocannabinoid System in Renal Physiology

### The Endocannabinoid System Within the Kidney

**Figure 1:**
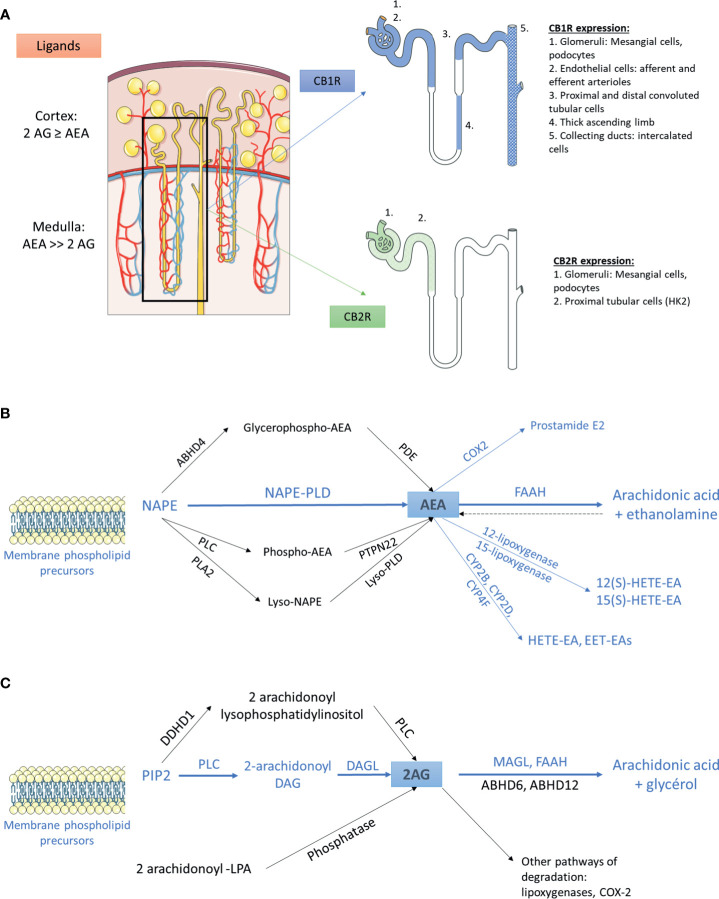
The endocannabinoid system within the kidney **(A)** Distribution of main endogenous ligands and cannabinoid receptors in healthy kidneys. **(B)** Biosynthesis and degradation of anandamide. The pathways that were demonstrated within the kidney are in blue. The other pathways are in black. **(C)** Biosynthesis and degradation of 2-arachidonoyl-glycerol. The pathways that were demonstrated within the kidney are in blue. The other pathways are in black. 2AG, 2-arachidonoyl-glycerol; ABHD, α/β-Hydrolase domain containing; AEA, anandamide; CB1R, cannabinoid receptor type 1; CB2R, cannabinoid receptor type 2; COX2, cyclooxygenase 2; CYP, cytochrome P450; DAG, diacylglycerol; DAGL, *sn*-1-specific diacylglycerol lipase; DDHD1, phosphatidic acid-preferring phospholipase A1; EET-EA, epoxyeicosatrienoic acid-ethanolamine; FAAH, fatty acyl amide hydrolase; HETE-EA, hydroxyeicosatetraenoic acid-ethanolamine; LPA, lysophosphatidic acid; MAGL, monoacylglycerol lipase; NAPE, N-arachidonoyl phosphatidyl ethanolamine; PDE, phosphodiesterase; PIP2, 2-arachidonoyl-phosphatidylinositol 4,5-bisphosphate; PLA2, phospholipase A2; PLC, phospholipase C; PLD, phospholipase D.

Little is known about the exact role of the ECS within the kidney, data being rather scarce and controversial (11) (**Figure 1**). As previously stated, ECS combines receptors, CB1R and CB2R, and ligands, the most prominent within the kidney being AEA and 2-AG. We will first describe what is known within the kidney about CB1R and CB2R expression, the pathways involved in endocannabinoid synthesis and the relationship between cannabinoid receptors and their ligands. Secondly, we will detail the role of the ECS in renal physiology and will mainly focus on the aspects that could be involved in the therapeutic management of CKD.

#### The Cannabinoid Receptors Within the Kidney

The cannabinoid receptors expression within the kidney was studied using transcriptional and protein approaches. Reverse transcription and polymerase chain reaction (RT-PCR) for *Cnr1*, the gene encoding for CB1R, western blot (WB) and immunohistochemical assays (IHC) demonstrated that CB1R was present in human and rodents (12–14, 27, 43–48). Assessing the specificity of proteins expression by IHC and WB, especially G-protein coupled receptor, needs adequate positive and negative controls to prevent improper interpretation of results (49, 50). Therefore, in the present review, we paid particular attention to the negative and positive controls used to confirm staining specificity (**Table 1**). Whereas CB1R expression was extremely low in healthy kidneys, we and others found than it was expressed in many cell types, especially in the vasculature (12, 26, 43). Its expression was also documented in other parts of the nephron including glomeruli (podocytes and mesangial cells) (13, 14, 43, 45) and tubular cells (27, 46, 48) (**Figure 1A**). Unlike CB1R, the expression of CB2R within the kidney remains controversial (52). Several groups failed to detect *Cnr2* mRNA, encoding for CB2R, and to demonstrate protein expression by WB and IHC (27, 46). Conversely, other groups found that CB2R may be expressed in normal renal cortex (43, 48, 53). In rats, *Cnr2* mRNA was found in mesangial cells (43). In human and mice, the pattern of CB2R staining by IHC was suggestive of podocyte labeling (53). Transcripts and proteins by WB were also found in human proximal tubular cell line (HK2), suggesting that CB2R could also be expressed in proximal tubules within the kidney (48) (**Figure 1A**). Taken together, these conflicting results suggest that CB2R is expressed at an extremely low level in normal kidneys. As previously stated, assessing the protein expression of G-protein by IHC is very tricky and antibodies specificity had to be carefully checked (49, 50). Transcriptional approach is helpful, especially when it targets a specific cell type. To date, data about the physiological role of CB2R within the kidney are lacking. Therefore, we will mainly focus on the role of CB1R.

**Table 1:** CB1R expression within healthy kidneys.

Structure	Species	Technical approaches	References
Whole kidney	Human	RT-PCR, IHC, WB	(27, 47, 51)
	Rats	RT-PCR, WB	(47, 48)
	Mice	RT-PCR, WB	(13, 44)
Endothelial cells	Human	IHC*	(12)
	Rats	RT-PCR, IHC^#^	(26, 43, 45)
Glomerular cells	Rats	RT-PCR, IHC (mesangial cells)	(43, 45)
	Mice	RT-PCR, IHC RT-PCR, IHC**/IF (podocytes)	(13, 14)
Tubular cells	Human	IHC (proximal and distal convoluted tubule cells; intercalated cells in the collecting ducts) RT-PCR, WB (HK2)	(27) (48)
	Rats	WB (TAL tubule cells)	(46)

*Mice cnr1^-/-^ tissue was used to assess the specificity of IHC staining (12).

**Specificity of the antibody binging was confirmed by disappearance of the staining when antibody was preabsorbed with a 10-fold excess of control peptide (26).

^#^Two-photon microscopy revealed a greater immunostaining for CB1R in afferent arterioles compared with efferent arterioles.

CB1R, cannabinoid receptor type 1; HK2, human proximal tubular cell line; IF, immunofluorescence; IHC, immunohistochemistry; RT-PCR, reverse transcription/polymerase chain reaction; TAL, thick ascending limb; WB, western blot.

#### The Endogenous Ligands of the Endocannabinoid System Within the Kidney

Whereas AEA was the first endogenous cannabinoid receptor ligand to be discovered (24), later evidence indicated that 2-AG was both the most abundant and the most efficacious endogenous natural ligand for the cannabinoid receptors in several tissues (54). Data about the main cannabinoid endogenous ligand within the kidney are contradictory: we and others found that 2-AG was the main endogenous ligand (12, 44), conversely to authors finding that AEA was more abundant than 2-AG (26, 43, 55, 56). This difference is possibly explained by the difference in the renal compartment analyzed in these studies: while kidney cortex exhibits similar or higher levels of AEA and 2-AG, AEA is the major endocannabinoid ligand of CB1R within the medulla.

AEA and 2-AG are eicosanoids that are synthesized on-demand from arachidonic acid containing phospholipids (57–59). The main source of AEA depends on the conversion of the membrane-bound N-arachidonoyl phosphatidyl ethanolamine (NAPE) (60–64). To date, the exact system responsible for NAPE’s synthesis within the kidney remains unknown (56). The next step is the conversion of NAPE to AEA. Several pathways have been described so far (54, 56, 60, 65). A direct mechanism involves the conversion of NAPE to AEA by the NAPE-specific phospholipase D (NAPE-PLD) (66). In addition, using NAPE-PLD^-/-^ mice, at least 3 groups demonstrated that multi-step NAPE-PDL-independent pathways were also able to convert NAPE to AEA, involving the formation of intermediate compounds which are thereafter converted to AEA (**Figure 1B**) (67–73). The NAPE-PDL could play a major role within the kidney as NAPE-PDL^-/-^ mice exhibited significantly higher NAPE level in kidney than wild-type mice (70). The presence of NAPE-PDL within the kidney was assessed by RT-PCR in immortalized epithelial cells derived from pig kidney proximal tubule (LLC-PK1 cells) (74). After biosynthesis, AEA is rapidly catalyzed by the fatty acid amide hydrolase (FAAH), an integral membrane serine hydrolase, to form arachidonic acid and ethanolamine (75, 76). Former studies demonstrated that FAAH hydrolyzed various N-acylethanolamines with a higher reactivity toward AEA and that FAAH was ubiquitously present in various tissues, including the kidney (75, 77, 78). Biochemical analysis revealed a higher relative FAAH activity in renal cortex than medulla, which could explain the high content of AEA in renal medulla (79). In addition to FAAH, other enzymes could also be involved in the degradation of AEA, such as cyclooxygenase type 2 (COX-2), lipoxygenases and cytochrome P450s. All these enzymes are expressed in the kidney (56, 80–84).

As for the biosynthesis of AEA, multiple pathways are involved in the biosynthesis of 2-AG (64, 85, 86) (**Figure 1C**). 2-arachidonoyl-phosphatidylinositol 4,5-bisphosphate (PIP2) is the main precursor of 2-AG. PIP2 is hydrolyzed by phospholipase C to form 2-arachidonoyl-diacylglycerol, which is further deacylated by *sn*-1-specific diacylglycerol lipase (DAGL) to yield 2-AG. 2-AG is then hydrolyzed to arachidonic acid and glycerol (64). This reaction can be catalyzed by several enzymes, including monoacylglycerol lipase (MAGL), FAAH, lipase α-β hydrolase (ABHD) 6 and ABHD12. The relative contribution of these enzymes differs among tissues and cells. Data about the pathways involved in biosynthesis and degradation of 2-AG in the kidney are scarce. To our knowledge, only Sampaio et al. demonstrated by RT-PCR that DAGL and MAGL were expressed in LLC-PK1 cells (74) (**Figure 1C**). Other endocannabinoids include N-acyl dopamine and 2-arachidonyl glyceryl ether, both of which binding strongly CB1R (87, 88). In addition to lipid compounds, some endogenous peptides could also bind to cannabinoid receptors. Recent studies found that hemopressin (HP), a peptide produced by the cleavage of the α1 chain of hemoglobin, behaved as an inverse agonist of CB1R (89, 90).

#### Relationship Between Ligands and the Cannabinoid Receptors

AEA and 2-AG bind both CB1R and CB2R, although with different affinities. Both ligands exhibit much higher affinity for CB1R than for CB2R: Ki of AEA were respectively 61-565 nM for CB1R *versus* 279-1940 nM for CB2R and Ki of 2-AG were respectively 58-472 nM for CB1R *versus* 145-1400 nM for CB2R (43, 54, 91). Numerous studies demonstrated that endogenous ligands also acted through non-cannabinoid receptors, the most studied being the transient receptor potential vanilloid type I channels (TRPV1) (92–100). TRPV1 belong to the transient receptor potential cation channel receptor family. Its activation was found to elicit renoprotection in rodent models of acute kidney injury (AKI) following ischemia/reperfusion (101–104). AEA could activate TRPV1, even though with a 20-fold lower affinity than CB1R (92, 95, 99). Experimental studies also demonstrated than AEA directly inhibited T-type calcium channels (100). Therefore, ECS is a complex system with numerous ligands lacking specificity and precisely decipher its role within the kidney remains challenging. However, as stated earlier, not only CB1R is the main cannabinoid receptor within the kidney, but also kidneys are enriched in AEA and in enzymes involved in its metabolism. As AEA had a better affinity for CB1R than for other receptors, many groups studying the ECS within the kidney used AEA infusion to explore the role of CB1R, although non-canonical effects of AEA may blur the results. Indeed, an effect of AEA through non-CB1R, such as TRPV1, could not be completely excluded. Many synthetic ligands were also developed to clarify the role of the ECS. These ligands behave as CB1R agonist or antagonist and exhibit various affinities for the cannabinoid receptors. Pharmacological properties of both endogenous and exogenous ligands that we cite in the present review, are summarized in **Table 2**. The most-studied pharmacological ligand is SR141716A (rimonabant), a CB1R inverse agonist which belongs to the first generation of CB1R antagonists. Rimonabant was briefly approved for clinical use but it was withdrawn from the market in 2007 due to severe central nervous side effects, mainly depression (29–36). Pharmacological studies demonstrated that rimonabant bound CB1R with much higher affinity than CB2R, Ki being respectively 1.98-12.3 nM for CB1R and 702-13200 nM for CB2R (108). However, like endogenous ligands, exogenous ligands also exert non-canonical effect and bound non-cannabinoid receptor as TRPV1 (93). However, directly targeting the endocannabinoid receptors seems the better approach to explore the therapeutic potential of the ECS since endogenous ligands are synthesized on-demand from arachidonic acid containing phospholipids through numerous pathways and bind both canonical than non-canonical receptors.

**Table 2:** Affinity of ligands for CB1R and CB2R.

Ligands	Ki for CB1R (nM)	Ki for CB2R (nM)	References
**Endogenous ligands**			
2-arachidonolyglycerol	58.3-472	145-1400	(43, 54, 91)
Anandamide	61-543	279-1940	(43, 54, 91)
**Exogenous ligands**			
MRI-1867^#^	1.2-8.0	>1000	(105)
WIN55,212-2	1.89-123	0.28-16.2	(106, 107)
SR141716A (rimonabant)	1.98-12.3	702-13200	(108)
JD5037	2	>1000	(109, 110)
AM6545	3.3	624	(38, 111)
Δ9-THC	5.05-80.3	3.13-75.3	(107)
AM251	7.49	2290	(112, 113)
(S)-SLV 319 (ibipinabant)	7.8	7.9	(114)
AM281	12	4200	(115)
(R)-Methanandamide	17.9-28.3	815-868	(116)
SR144528	400	0.6	(117)
AM1241	580	7	(118)
JWH-133	677	3.4	(119)
AM630	5152	31.2	(120)

CB1R, cannabinoid receptor type 1; CB2R, cannabinoid receptor type 2; Ki, inhibition constant; THC, tetrahydrocannabinol.

^#^MRI-1867 is a dual CB1R/inducible NOS antagonist.

### The Vascular Functions of the Endocannabinoid System Within the Kidney

**Figure 2:**
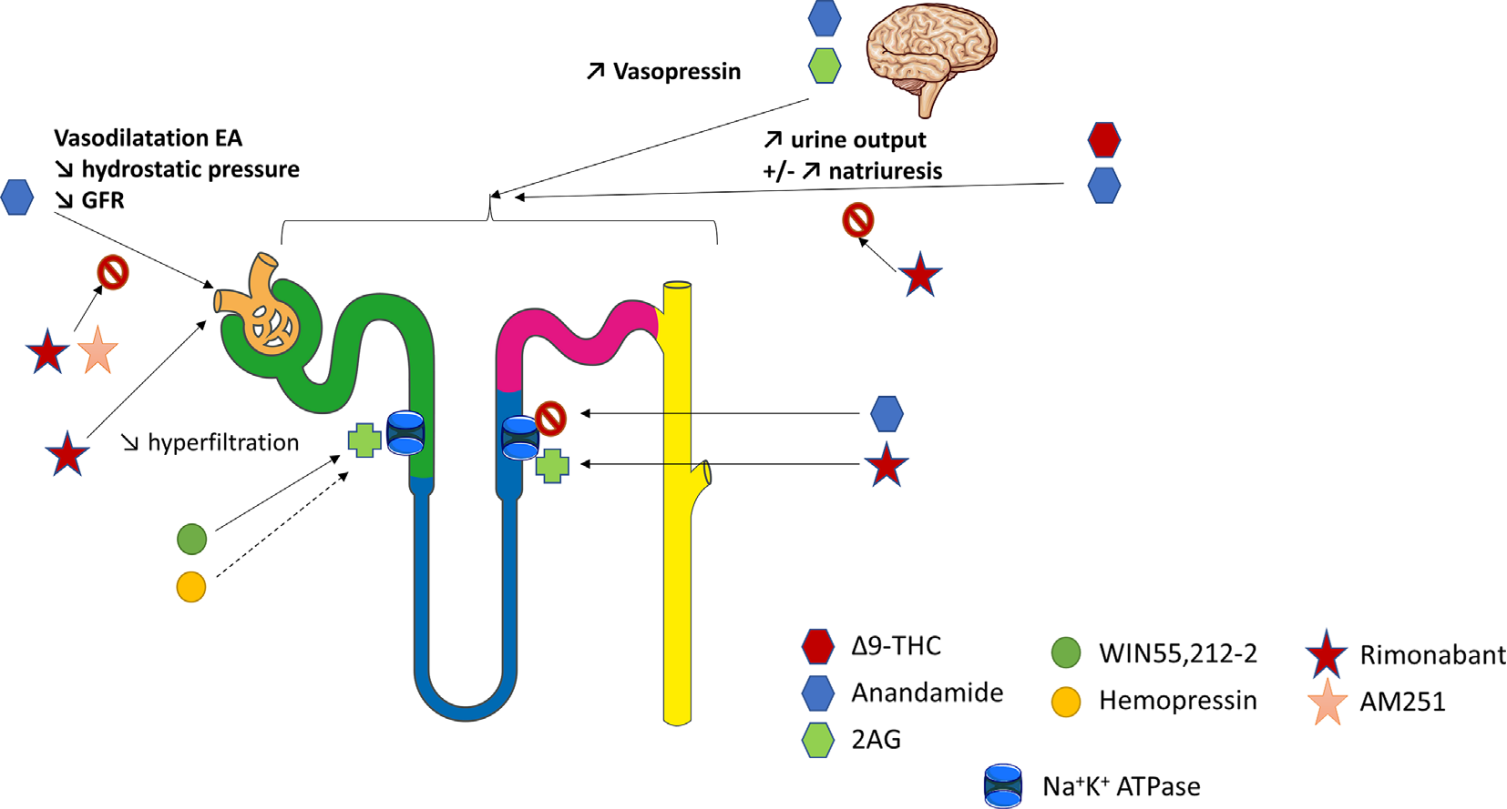
The physiological role of CB1R within the kidney. Overview of the current knowledge of the role of CB1R in renal vasculature, tubular cells and blood pressure regulation. The figure has taken intp account both *in vitro* and *in vivo* experiments. 2AG, 2-arachidonoyl-glycerol; EA, efferent arterioles; GFR, glomerular filtration rate; THC, tetrahydrocannabinol.

In the normal kidney, CB1R expression is more prominent in the vasculature where it contributes to the regulation of renal hemodynamic (12, 26, 43). Deutsch et al. demonstrated that renal endothelial cells express mRNA for CB1R, and selectively bind AEA (43). In rats, AEA injection induced afferent arterioles vasodilation through CB1R binding by increasing nitrogen monoxide (NO) production since this response was inhibited by SR141716A (43) (**Figure 2**). The arteriolar responses to AEA were also assessed *ex vivo* by the blood-perfused juxtamedullary nephron technique (26). Whereas high doses of AEA significantly increased the diameter of both afferent and efferent arterioles, lower doses were responsible for predominant efferent arteriolar dilation, inducing a fall in glomerular filtration rate (GFR). In this model, selective antagonism of CB1R by AM251 (**Table 2**) attenuated the afferent arteriolar response to AEA and inhibited the efferent arteriolar response to AEA. Conversely, AM281, a nonselective antagonist of cannabinoid receptors inhibited responses to AEA in both afferent and efferent arterioles. Taken together, these results suggest that efferent arterioles are more sensitive to AEA and that AEA causes greater efferent arteriolar dilation *via* CB1R, which is consistent with a decreased hydrostatic pressure in glomerular capillaries and therefore a decrease in GFR (26, 56). However, the best way to fully decipher the vascular role of CB1R within the kidney would be to study endothelial cells specific CB1R knockout rodents. To our knowledge, such studies are lacking nowadays. As both endogenous and pharmacological ligands may also act through non-cannabinoid receptors, afferent arteriolar dilatation by high doses of AEA could therefore be amplified by non-cannabinoid effect of AEA. In CKD, the increase of capillary pressure participates to nephron reduction. Thus, the decrease of capillary pressure possibly mediated by CB1R activation could be beneficial in CKD. Conversely, an acute drop of capillary pressure could be damaging in normal kidneys, similarly to what is observed during RAS inhibition. Therefore, a better understanding of the vascular effects of CB1R and of its inhibition is mandatory if CB1R inhibition become a therapeutic target in CKD.

### The Role of the Endocannabinoid System in Tubular Cells

The ECS could also be involved in tubular functions, especially in the regulation of water and sodium balance (**Figure 2**). Thus, it could offer therapeutic perspectives in the management of hypertension (HTN) or conversely promote sodium retention. Former studies investigating the renal effects of marijuana shown that Δ9-THC, binding both CB1R and CB2R with similar affinity (**Table 2**), increased urine production (121, 122). This effect was first thought to be centrally mediated (123–126). Indeed, both AEA and 2-AG, released from the hypothalamus, act directly on the hypophysis to modulate glutaminergic and GABAergic pathways that induce hormone release such as vasopressin (125, 126). However, recent data shown that a peripheral modulation could also be involved (46, 79, 127–129). As mentioned above, several studies confirmed that tubular cells express the main components of ECS, including CB1R and possibly CB2R as well as the enzymes for biosynthesis and degradation of their ligands (74, 130). However, not only the physiological role of CB1R in tubular cells, but also the mechanism responsible for an aquaretic and/or diuretic effect of ECS, remain controversial. In rat kidneys, the effect of oral THC administration on urine and Na^+^ output was comparable to the thiazide diuretic, hydrochlorothiazide (122). More recently, Ritter et al. reported a natriuretic effect of AEA (79, 130). They found that AEA infusion into the mouse renal medulla increased both sodium excretion and urine output (79, 130). The authors initially reported that a non-canonical CB receptor-based mechanism could be involved (79). Indeed, celecoxib, a selective COX-2 inhibitor, blocked the effects of AEA. Therefore, the action of AEA could be mediated indirectly by its COX-2 metabolite, prostamide E2. Their hypothesis was reinforced by the fact that infusion of prostamide E2 mimicked the effects of AEA in stimulating natriuresis and diuresis in mice. More recently, Ritter et al. found that rimonabant also blocked the effects of AEA, suggesting a CB1R-dependent effect of intramedullary AEA on flow rate and natriuresis (130). Using *ex vivo* experiments on isolated mouse kidney thick ascending limb (TAL) tubule cells, they demonstrated an inhibitory effect of AEA on the Na^+^/K^+^-ATPase activity (130). Another team measured oxygen consumption in rat TAL suspensions to monitor the effects of AEA (46). AEA reduced oxygen consumption in a concentration-dependent manner. WIN55,212-2 (WIN), a non-selective lipid cannabinoid agonist binding both CB1R and CB2R with a better affinity for CB2R (**Table 2**), also reduced oxygen consumption whereas rimonabant blocked the effect of AEA. Conversely, both the CB2R-selective agonist JHW-133 and the CB2R antagonist AM630 were ineffective (**Table 2**). The authors concluded that AEA inhibited TAL Na transport-related oxygen consumption by activating CB1R and NO stimulation, which, in turn, could block apical transporters and therefore be associated with a natriuretic effect (**Figure 2**).

Conversly, other authors found that ECS was involved in sodium retention (26, 128). In rats, intramedullary infusion of methanadamide (**Table 2**), a stable analogue of AEA, increased diuresis without any effect on natriuresis (128). Similarly, intrarenal AEA injection did not alter urinary sodium excretion (26). These results were enhanced by an *in vitro* study on LLC-PK1 cells in which the authors have shown that ECS could decrease renal sodium reabsorption by directly stimulating Na^+^/K^+^-ATPase (74). WIN was responsible for an early and sustained increase of Na^+^/K^+^-ATPase activity whereas HP displayed a biphasic effect leading to an early significant increase in the Na^+^/K^+^-ATPase activity and acting as a potent inhibitor of the pump after 15 min. The CB1R antagonist AM251 (**Table 2**) was used to demonstrated that the fast effect of both WIN and HP on Na^+^/K^+^-ATPase activity was independent of CB1R whereas the slow one was dependent on CB1R. Different signaling pathways were stimulated by WIN and HP, respectively protein kinase C (PKC) and protein kinase A (PKA), resulting in different effects on the Na^+^/K^+^-ATPase (74). Sampaio et al. also studied the role of ECS in an acute model of experimental ischemia reperfusion, both *in vivo* in Wistar rats and *in vitro* using antimycin A treatment on LLC-PK1 epithelial cells (131). Antimycin A induced ATP depletion and was therefore used as an *in vitro* model of ischemia reperfusion. The authors demonstrated that the ECS and Na^+^/K^+^ ATPase were downregulated 24 hours after the ischemia reperfusion. *In vitro*, they found that the downregulation of Na^+^/K^+^ ATPase could be reversed by WIN in a CB1R dependent manner (131).

Thus, the role of ECS in tubular cells within the kidney remains largely unknown. Either CB1R may promote natriuresis and its therapeutic inhibition will give HTN and sodium retention (like endothelin-1 antagonists), or conversely CB1R inhibition could cause natriuresis and be beneficial in HTN treatment. Effects of AEA may depend on dose, route of injection, cells (TAL *versus* renal proximal tubular epithelial cells, RPTC). Different pathways and sodium channels are involved in sodium reabsorption throughout the various tubular segments within the nephron and AEA may act through different mechanisms. Once again, as not only endogenous ligands but also pharmacological compounds could exert CB1R-independent effects, the best way to fully decipher the tubular role of CB1R within the kidney would be to study tubular-specific CB1R knockout rodents. As explained earlier for CB1R and vascular function, a better understanding of CB1R role in water and sodium balance is paramount if CB1R become a new therapeutic tool in CKD.

### The Role of the Endocannabinoid System on Blood Pressure Regulation

Both the vascular functions of the endocannabinoid system within the kidney and the role of the ECS in tubular cells highly suggest an involvement of the ECS on blood pressure (BP) regulation, which represents a major factor in the management of CKD progression (1) (**Figure 2**). Former studies demonstrated that chronic use of Δ9-THC not only increased urine production, but also decreased heart rate and BP, both in human and animals (132–135). The same effect was observed in rats after intravenous perfusion of Δ9-THC (133, 136). A triphasic response was described involving a vagal-mediated fall in blood pressure (phase I), followed by a brief pressor effect (phase II) and finally a prolonged hypotensive effect (phase III) (136–138). Multiple mechanisms seemed to be involved, both central, mainly through cardiovascular centers of brainstem and hypothalamus, and peripheral (58). Central mechanisms of BP regulation by the ECS were deciphered first. Several studies demonstrated that activation of CB1R was responsible for a decrease of sympathetic activity leading to neurologic-mediated hypotension (132, 133, 136, 139). AEA could modulate the baroreflex through activation of CB1R within the nucleus tractus solitarius, possibly by modulating effectiveness of GABA and/or glutamate neurotransmission (140). CB1R also participates to the pressor effect of angiotensin II (Ang II) infusion in the brain (58, 141).

Next, peripheral mechanisms of BP regulation by the ECS were also identified. Cardiac activation of CB1R exerted negative chronotropic and inotropic effects independently of the central nervous system, therefore promoting a decrease in BP (58, 142). In addition to direct cardiodepressant effects, studies highlighted a vasodilatory effect of the ECS ligands in aorta and coronary arteries, through both CB1R-dependent and independent pathways that could also participate to a decrease in BP (143–148). Interestingly, *Cnr1^-/-^* mice have the same baseline BP than their wild-type littermates (21, 149). Treatment of normotensive rats and mice with the CB1R antagonist SR141716A alone has also little effect on BP (133, 136), as well as inhibition of AEA transport (150). Taken together, these results suggest that, under baseline conditions, CB1R is not tonically active in vessels and do not participate to baseline BP regulation (56). Conversely, effects of the ECS through CB1R modulation were observed in 3 experimental models of hypertension (HTN): the spontaneously hypertensive rat (SHR) model, the Dahl salt-sensitive and salt-resistant rat model and chronic Ang II infusion- induced hypertension in Sprague Dawley rats (151–153). Once again, BP of the normotensive control rats was not altered by either CB1R agonists (Δ9-THC, AEA) or CB1R antagonists (SR141716A, AM251) which were used in these studies (151–153). Bátkai et al. demonstrated the involvement of CB1R in BP regulation: the CB1R antagonists SR141716A and AM251 aggravated HTN in SHR whereas URB597, an inhibitor of FAAH, reduced BP in SHR and in the chronic Ang II infusion induced HTN (151). Conversely, in SHR, not only the CB2R antagonist SR144528 (**Table 2**) did not affect BP, but also the TRPV1 antagonist capsazepine did not alter BP decrease by AEA intra-venous administration (151). The mechanisms of BP reduction in these models were attributed to the effects of endocannabinoids on cardiac contractility and vascular resistance. Endocannabinoids tonically suppressed cardiac contractility in hypertension and could normalize blood pressure by enhancing the CB1R-mediated cardiopressor and vasodilator effects of endogenous AEA (151). Furthermore, an interaction between the RAS and the ECS was identified in rodent peripheral arterioles. Ang II stimulated vascular endocannabinoid formation, which attenuated its vasoconstrictor effect, suggesting that endocannabinoid release from the vascular wall and CB1R activation reduced the vasoconstrictor and hypertensive effects of Ang II (154, 155).

CB1R may also modulate the Ang II pressor effect by its action on sodium and water balance. As described earlier, CB1R may be involved in the regulation of salt and water balance (46, 79, 122, 127–130). Furthermore, Ang II type 1 receptors (AT1) are largely expressed within the kidney. A recent review highlighted that the juxtaglomerular and tubulo-interstitial areas were conserved expression sites for AT1 across species and could represent the main target sites for Ang II in adult human and rodent kidneys (156). In addition, as demonstrated using tissue-specific AT1a knockout mice (157), AT1 receptors within the kidney (and not in the heart) promote the pressor response to Ang II. Several studies found interactions between AT1 and the ECS in numerous organs and systems (58, 141, 154, 155). On the molecular basis, the signal integration between CB1R and AT1 could be due to AT1R–CB1R heteromerization (15, 158). AT1R–CB1R heteromerization was first demonstrated in Neuro2A cells, a neuroblastoma cell line that contains endogenous CB1R (158). In hepatic stellate cells from ethanol-administered rats in which CB1R is upregulated, Rozenfeld et al. found a significant upregulation of AT1R–CB1R heteromers and enhancement of angiotensin II-mediated signaling. Moreover, CB1R inhibition by rimonabant prevented angiotensin II-mediated mitogenic signaling and profibrogenic gene expression (158). Therefore, the interaction with CB1R could confer new signaling properties to AT1R and enhanced responsiveness to Ang II. Jourdan et al. found that losartan, an AT1R antagonist, attenuated diabetic nephropathy in rats by downregulating the expression of CB1R in podocytes, reinforcing this hypothesis (15). Thus, a role for the ECS and CB1R within the kidney in the physiopathology of HTN cannot be excluded and it deserves to be studied, especially because a large proportion of CKD patient, who could be treated by CB1R antagonists, may suffer from HTN.

## The Role of Cannabinoid Receptors in Chronic Kidney Diseases and Renal Fibrosis

### CB1R, and Not CB2R, Is the Main Actor of the Endocannabinoid System Promoting Renal Fibrosis

Fibrogenesis is a multifactorial process leading to excessive extracellular matrix deposition, regardless of the organ and cause. Several studies demonstrated that endocannabinoids acting *via* CB1R promote fibrosis and that CB1R blockage reduces fibrogenesis in many organs including the liver (159–161), the heart (162), the lungs (163, 164) and the skin (165). As for the kidneys, CB1R is also an active direct player in renal fibrogenesis. This was first suggested by a microarray analysis performed by our team (12). Using the unilateral ureteral obstruction (UUO) experimental model, a rapid and reproducible model of renal fibrosis in mice, we compared the gene expression profile of fibrotic kidneys with contralateral undamaged kidneys by microarrays analysis (12). As expected, we found many overexpressed genes well-known to be involved in renal fibrosis, such as *tgfb, mmps* and *timp.* Remarkably, *Cnr1* was among the ten most significantly upregulated genes during renal fibrosis (12). In addition, a growing body of evidence highlighted that CB1R could also be a new therapeutic target to prevent renal fibrosis in both metabolic and non-metabolic diseases, as we will discuss next in our review (11–18). Conversely, the role of CB2R during renal fibrosis remains largely unknown, studies being scarce and contradictory. Several studies elicited that CB2R blockage promoted fibrosis in the liver (166), the heart (167) and the skin (168), whereas CB2R agonists decreased fibrogenesis (166–168). Surprisingly, Zhou et al. found that CB2R overexpression could promote renal fibrosis (169, 170). We and others found opposite results with a protective effect of CB2R activation in various models of CKD (12, 53, 171). During UUO, we demonstrated that CB2R antagonist alone (SR144528) increased the development of renal fibrosis whereas CB2R agonist (JWH 133) alone blunted it (12). However, neither SR144528 nor JWH133 further reduced the development of fibrosis when compare with CB1R pharmacological blockade by rimonabant (12). Barutta et al. demonstrated that CB2R was downregulated in kidney biopsies from patients with advanced diabetic nephropathy (53). They also found that AM1241, a CB2R agonist (**Table 2**), ameliorated albuminuria, podocyte protein downregulation, and glomerular monocyte infiltration, without affecting early markers of fibrosis. In addition, AM1241 reduced CC chemokine receptor 2 (CCR2) expression in both renal cortex and cultured podocytes, suggesting that CB2R activation may interfere with the deleterious effects of monocyte chemoattractant protein-1 (MCP-1) signaling (53). In streptozotocin (STZ)-induced diabetic mice, the genetic deletion of CB2R exacerbated the downregulation of podocin and nephrin, mesangial expansion, monocyte infiltration, and reduced renal function overexpression of extracellular matrix. The deletion of CB2R also enhanced the expression of fibrosis marker (171). Given the contradictory results of the various studies, the exact role of CB2R in renal fibrosis needs to be further explored. To date, there is no ongoing clinical study investigating the effect of CB2R on renal function. We will therefore focus on the therapeutic potential of CB1R inhibition in renal fibrosis.

### CB1R Promotes Renal Fibrosis Associated With Metabolic Disorders

**Figure 3:**
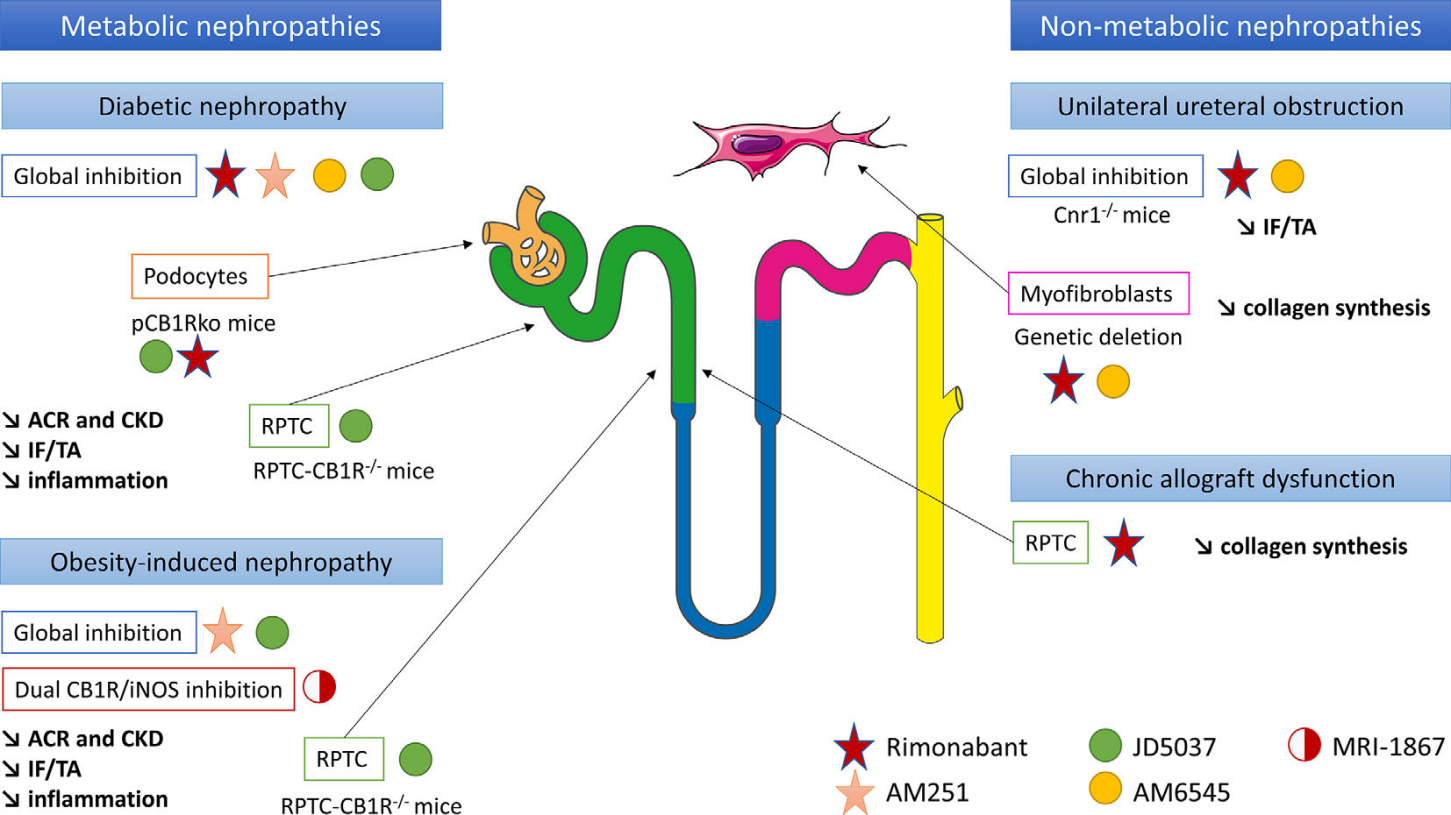
CB1R inhibition could be a new therapeutic target in chronic kidney diseases and renal fibrosis, in both metabolic and non-metabolic nephropathies. Both *in vitro* and *in vivo* experiments have been taken into account. ACR, urinary albumin-to-creatinine ratio; CB1R, cannabinoid receptor type 1; CKD, chronic kidney disease; IF/TA, interstitial fibrosis and tubular atrophy; iNOS, inducible nitric oxide synthase; pCB1Rko, podocyte-specific deletion of CB1R; RPTC, renal proximal tubular cells.

The role of CB1R in renal fibrosis was first documented during diabetes and metabolic syndrome (13, 15–18, 45) (**Figure 3**). We and others demonstrated that renal CB1R expression was increased in diabetic nephropathy, both in humans (12, 45) and rodents (14, 15, 17, 18). Numerous studies demonstrated that CB1R blockade improves multiple parameters involved in metabolic syndrome and diabetes, such as waist circumference, glycemia, glycated hemoglobin, HDL and LDL cholesterol and triglycerides (13, 29, 30, 45). Both global genetic CB1R inactivation and pharmacological blockade by first generation CB1R antagonists dramatically improves metabolic parameters (13, 14, 45) (**Figure 3**). Indeed, CB1R inhibition may protect the kidney from metabolic induced fibrosis and injury by improving systemic metabolic parameters. Thus, experimental studies in rodents demonstrated that rimonabant and AM251, belonging to the first generation CB1R antagonists with BBB crossing, protect the kidney from the development of albuminuria, CKD, renal fibrosis, glomerulosclerosis and renal inflammation in diabetes (13, 14, 45). However, recent studies also exhibited a role of peripheral CB1R in metabolic nephropathies. Udi et al. highlighted that CB1R regulated obesity-induced CKD by acting on RPTC (16). Indeed, specific deletion of CB1R in RPTC (RPTC-CB1R^-/-^) did not prevent obesity in mice, but significantly reduced the obesity-induced lipid accumulation in the kidney as well as renal dysfunction, urinary albumin-to-creatinine ratio (ACR), inflammation, and renal fibrosis. Deciphering the pathways involved in this experimental model, the authors found that CB1R acted through the Gα_i/0_-PKA axis. Therefore, CB1R blockade mediated the downstream activation of the tumor suppressor liver kinase B1 (LKB1), which modulates AMP-activated kinase (AMPK) activity by inducing its phosphorylation. AMPK inactivates acetyl-CoA carboxylase (ACC) by its phosphorylation, increasing fatty β-oxidation in renal proximal tubules. *In vitro*, CB1R blockade by JD5037 increased fatty acid β-oxidation in proximal tubular cells and protected the kidney from obesity-induced fibrosis.

Similarly, the peripheral role of CB1R during renal fibrosis in diabetic nephropathy was recently documented. The first data supporting a peripheral role of CB1R in diabetic nephropathy and an antifibrotic effect independent from the improvement of systemic metabolic parameters were reported by Janiak et al. (45). Rimonabant, the most-known first generation CB1R antagonists crossing BBB, significantly reduced body weight, blood glucose and improved lipid profile in rodents (14, 45). Comparing obese Zucker rats receiving either rimonabant or vehicle for 12 months to pair-fed but untreated group of obese rats, Janiak et al. demonstrated that rimonabant significantly reduced proteinuria and renal failure as well as tubulo-interstitial fibrosis and glomerulosclerosis compared to per-fed animals (45). In addition, in cultured podocytes *in vitro*, high glucose stimulation increased CB1R expression whereas rimonabant abolished high-glucose-induced up-regulation of collagen and plasminogen activator inhibitor-1 synthesis, also suggesting that CB1R blockade protected against renal injury through both metabolic and antifibrotic effects in type 2 diabetic nephropathy (14). A study of Barutta et al. reinforced this hypothesis (13). Whereas CB1R blockade by AM251 in STZ-induced diabetic mice had no effect on body weight, blood glucose and BP, it significantly reduced ACR and prevented down-regulation of nephrin, podocin and zonula occludens 1 (ZO-1). Because histological lesions, including renal fibrosis, were very mild, a possible beneficial effect of CB1R blockade preventing overt lesions in diabetic nephropathy could not be demonstrated (13).

The same results were observed using JD5037 and AM6545, peripherally restricted CB1R antagonists that did not cross BBB. Whereas JD5037 and AM6545 were quite ineffective in controlling blood glucose and glycated hemoglobin levels (13, 15, 17, 18), they significantly improved renal parameters, especially renal function, ACR and renal inflammation (13–15, 17, 18, 39, 40, 45) in Zucker diabetic fatty (ZDF) rats (13, 15), STZ-induced diabetic mice and Akita diabetic mice (17). Several pathways seem to be involved. In ZDF rats, Barutta et al. found that JD5037 improved glomerular filtration and reversed albuminuria, activation of the RAS, oxidative/nitrative stress and podocyte loss (13). However, no evidence of chronicity (glomerulosclerosis, interstitial fibrosis or tubular atrophy) was observed at the point (90 days) at which the cohort was sampled for histopathological analysis, preventing any conclusion regarding a decrease in overt renal diabetic lesions (13). Thus, these results suggest that improvement mainly depends on renal hemodynamic in this model. Another work published by Jourdan et al. in ZDF rats reinforces this hypothesis (15). Indeed, not only the authors demonstrated that CB1R signaling in podocytes increased RAS activity, which was down-regulated by CB1R blockade, but also they found a direct link between AT1 activation and CB1R expression *in vivo* in ZDF rats (15). In podocytes *in vitro*, they demonstrated a cross talk between reactive oxygen species, angiotensin II and slit-diaphragm protein expression such as nephrin (15). In STZ-induced diabetic nephropathy, some authors extended the delay before analysis to allow time for renal lesions, especially renal fibrosis, to develop (18, 39, 40). After 14 weeks, Barutta et al. found that AM6545 reduced mesangial expression, upregulation of glomerular fibronectin and collagen deposition in both the mesangial and the interstitial area (39). AM6545 also significantly reduced down-regulation of nephrin and podocin (40) and prevented diabetic-induced downregulation of markers of anti-inflammatory M2 macrophages which play a key role in resolving inflammation and promoting repair (39, 40). To precisely assess the role of CB1R podocyte expression in diabetic nephropathy, Jourdan et al. reported a mouse model with podocyte-specific deletion of CB1R (pCB1Rko) STZ-induced diabetic nephropathy (18). Whereas hyperglycemia was similar in both pCB1Rko and their wild-type littermate, pCB1Rko mice exhibited less ACR, podocytes loss, tubular dysfunction and interstitial fibrosis. These parameters were mediated in part by podocyte-derived endocannabinoids acting *via* CB1R on proximal tubular cells (18). In addition, the role of CB1R expression in RPTCs in diabetic nephropathy was studied by Hinden et al. in STZ-induced diabetic mice using RPTC-specific deletion of CB1 (RPTC-CB1R^-/-^) (17). RPTC-CB1R^-/-^ mice developed only mild diabetes manifesting by modest hyperglycemia and were almost completely protected from the development of diabetic nephropathy compared to their littermate counterparts, with a better renal function and a decrease of ACR, kidney inflammation and tubule interstitial fibrosis. These effects were due to a downregulation of glucose transporter 2 (GLUT2) expression in RPTC. Indeed, GLUT2, localized in RPTCs, affects the basolateral efflux of glucose from the tubular cell back to the circulation (172, 173). Its expression is increased in humans with diabetes and in murine models of diabetes (174–177). Hyperglycemia increases tubular GLUT2 expression and shifts its localization from basolateral membrane to the apical/brush border membrane, contributing to increase glucose reabsorption (174, 178). CB1R blockade was related with the disruption of glucose-induced Ca^2+^-dependent PKC -β1 activation which, in turn, modulated GLUT2 translocation and/or expression in RPTC, through the G_q/11_ signaling. *In vitro*, CB1R stimulation mimics these effects whereas blockade of CB1R by JD5037 blunts these effects (18).

Thus, all these studies documented that the beneficial effect of CB1R blockade was not only due to its role on metabolic parameters, but also through a direct action on renal cells (podocytes and RPTCs) independently of metabolism. These studies also demonstrated that different cellular pathways are involved in renal fibrogenesis during metabolic nephropathies.

### CB1R Activation Promotes Non-Metabolic Renal Fibrosis

So far, only our group studied the role of CB1R in an experimental model of non-metabolic renal fibrosis *in vivo* in mice (12) (**Figure 3**). Whereas CB1R expression was low in normal kidneys, it was increased both in metabolic (diabetic nephropathy) and non-metabolic (acute interstitial nephritis, IgA nephropathy) nephropathies (12). Furthermore, there was a significant negative correlation between CB1R expression and kidney function. In mice in the UUO model, both the pharmacological blockade by rimonabant or by AM6545 and the genetic disruption of CB1R profoundly reduced the development of renal fibrosis. This effect was mainly due to a direct paracrine/autocrine role of CB1R in myofibroblasts, which are the final effector cells in renal fibrogenesis. We also found that upon transforming growth factor β (TGFβ) stimulation, renal myofibroblasts expressed CB1R and secreted endocannabinoid ligands, whereas CB1R blockade reduced collagen synthesis (12). During chronic kidney allograft dysfunction (CAD) in humans, we found that CB1R was highly expressed in tubular cells (28). CAD remains the first cause of graft loss (179) and corresponds to the progressive and inevitable impairment of renal graft function. It is a multifactorial and complex process, in which a lot of immunological and non-immunological causes are involved (180–184). Recently, antibody-mediated rejection as emerged as one of the major causes of CAD (184–186). During CAD, the functional renal tissue is replaced by extracellular matrix proteins, mainly collagens, leading to both interstitial fibrosis and tubular atrophy. Other histological damages associated glomerulosclerosis and vascular intimal hyperplasia (187). We highlighted that CB1R expression significantly increased in the first three months after kidney transplantation, and it remained stable thereafter. CB1R expression in preimplantation kidney graft biopsies was higher than CB1 expression we previously found in normal kidneys (23% ± 15% *versus* 6.5 ± 4.8%) and was not correlated with renal fibrosis at this particular time-point (12, 28). Therefore, we hypothesized that the high level of CB1R expression in preimplantation biopsies could be a consequence of cold ischemia-induced acute tubular necrosis and could be predictive for the development of further renal graft fibrosis. Next, we found a significant positive correlation between CB1R expression and renal graft fibrosis at 3 months post-transplantation (28) (**Figure 3**). Moreover, patients with stable renal fibrosis during the first-year post-transplantation tended to have lower increase in CB1R expression than patients in whom renal fibrosis increased. Thus, CB1R could promote the early steps of the development of CAD or at least be a marker of renal fibrosis. *In vitro*, we found that an anticalcineurin treatment by tacrolimus significantly induced mRNA and protein expression of CB1R, concomitantly to collagen up-regulation. Administration of rimonabant blunted collagen synthesis. The impact of cannabinoid system modulation during CAD as well as the cellular and molecular pathways involved, remains to be clarified.

Overall, we found evidence of a profibrotic role of CB1R in experimental mice models, human renal biopsies and *in vitro* studies. However, the respective contribution of tubules and myofibroblasts remains to decipher. In the UUO model, we found that whereas CB1 expression was drastically increased in the tubules, interstitium and glomeruli, CB1R blockade significantly reduced the increase of renal fibrosis through a direct paracrine/autocrine role of CB1R in myofibroblasts with no strong evidence for a direct role of CB1R expressed in tubules (12). During CAD, we found that CB1R was highly expressed in tubular cells and that CB1R blockade reduced collagen synthesis by tubular cells. Thus, identifying the respective role of CB1R in tubules and myofibroblasts during non-metabolic renal fibrosis remains to be established. The best way to fully answer the question would be to study cell-specific CB1R knockout rodents. The cellular pathways involved in non-metabolic renal fibrosis mediated by CB1R also remain to be elucidated.

## CB1R Inhibition: A New Promising Therapeutic Target in Chronic Kidney Diseases

As presented earlier, experimental data highlighted the therapeutic potential of CB1R blockade in renal fibrosis and CKD, regardless of the cause (metabolic or not) (12, 13, 15–18, 45). Therefore, there is a growing interest for the industry to develop CB1R inhibitors with no BBB passage to improve tolerance compared with rimonabant (188).

### The First Generation CB1R Antagonists: Rimonabant

Rimonabant was the only CB1R antagonist approved for clinical use in obesity. However, clinical trials with rimonabant mainly focused on metabolic syndrome and patients with CKD were excluded from these studies (29–36). The RIALTO study (NCT00458081) aimed to assess the effect of rimonabant on microalbuminuria in patients with metabolic syndrome. Assessment of glomerular filtration rate was a secondary objective of the study. Due to central nervous system adverse events, especially an important risk of severe depression, rimonabant was withdrawn in 2008, before the end of the RIALTO study. The study was also prematurely terminated. Therefore, to date, no clinical trial investigated the effect of CB1R inhibition on CKD course in diabetes and obesity.

### The Second Generation CB1R Antagonists: Peripherally Restricted CB1R Antagonists

Multiple peripherally CB1R antagonists, without central nervous system side-effects, were developed these past 10 years (37). As discussed earlier in the present review, experimental studies demonstrated that some molecules, such as AM6545 or JD5037, could be useful to prevent renal fibrosis and therefore be good candidates to be part of the therapeutic tools against CKD (12, 16, 18, 37–39). AM6545 is a non-brain-penetrant neutral CB1R antagonist, based on the rimonabant template but with an alkynyl chain off the 4-aryl group. It retains high affinity and selectivity for CB1R with Ki of 3.3 nM for CB1R, which is similar to Ki of rimonabant (38). Importantly, AM6545 displays markedly reduced brain penetrance (38, 111). AM6545 significantly reduced diabetic-induced albuminuria in diabetic mice (39, 40) as well as collagen deposition in both the mesangial and the interstitial area (39). Using UUO, a non-metabolic experimental model of renal fibrosis, we demonstrated a 25 to 60% reduction of renal fibrosis in AM6545 compared to vehicle treated mice.

While the rimonabant template was the most common choice for developing peripherally restricted CB1R antagonists, the Jenrin group developed JD5037 from ibipinabant, another first generation CB1R antagonist (109, 189). It was found that modifications of the central N-methyl group of ibipinabant with polar pendants provided analogs that followed physicochemical guidelines for diminishing blood brain barrier penetration. JD5037 contained pendants of N-substituted valinamide and exhibited 15-fold greater affinity for CB1R (IC50 1.5 nM) than ibipinabant (109). Preclinical toxicity studies were performed with high doses of JD5037 in rats and dogs (respectively 150 mg/kg/d and 20-75 mg/kg/d) without observed adverse effects. Experimental studies in rodents demonstrated that JD5037 was effective in mitigating both diabetic nephropathy by blocking overactive CB1R in podocytes (15) and regulating renal GLUT2 in proximal tubular cells (18) and obesity-induced nephropathy by blocking overactive CB1R in proximal tubular cells (16). The CB1R blockade by JD5037 increases fatty acid β-oxidation in proximal tubular cells and protects the kidney from obesity-induced dysfunction and injury. To date, data about the effect of JD5037 in non-metabolic renal fibrosis are lacking. Therefore, peripherally restricted CB1R antagonists may be good candidates for slowing CKD progression, especially during metabolic nephropathies, and experimental data are encouraging to move up forward clinical trials in the short term. JD5037 is now developed under the name of CRB-4001 (Corbus Pharma) and a Phase II study in patients with non-alcoholic steatohepatitis is underway. Recently, Liu et al. described a peripheral CB1R antagonist (MRI-1891) highly biased toward inhibiting CB1R-induced β-arrestin-2 recruitment over G-protein activation. In mice, MRI-1891 reduced food intake, body weight and obesity-induced muscle insulin resistance *via* β-arrestin-2 signaling (42).

### The Third Generation CB1R Antagonists: Peripherally Restricted Dual-Target CB1R Antagonists

Recently, hybrid CB1R antagonists were designed to improve therapeutic efficacy for instance in liver fibrosis (105, 190) and in pulmonary fibrosis (164). Experimental studies demonstrated the usefulness of peripherally restricted hybrid CB1R/inducible nitric oxide synthase (iNOS) antagonists during CKD (37, 41). Indeed, both CB1R and iNOS are increased during CKD (51). iNOS overactivity was found to contribute to tubular dysfunction in obesity-induced CKD in mice (191). Interestingly, iNOS overactivity was also found to contribute to renal fibrosis in UUO, a non-metabolic experimental model of renal fibrosis (192). Udi et al. demonstrated that MRI-1867, a dual CB1R/iNOS antagonist, was more efficient than JD5037 or an iNOS inhibitor (1400W) alone to prevent obesity-induced kidney injury, inflammation, fibrosis and kidney dysfunction in mice (51). Similarly, data about the effect of MRI-1867 on non-metabolic renal fibrosis are lacking.

Another hybrid molecule could also be an activator for the secondary target, such as AMPK activator (37). Supplemental experimental data are mandatory to move forwards to therapeutics. No phase II trial is ongoing yet. Assessing safety and tolerability, especially central nervous system, would be the first stage of clinical development in human pathologies.

### CB1R Blocking Antibodies

Blocking antibodies targeting CB1R is another interesting approach to prevent central nervous system side-effects induced by CB1R inhibition. The Bird Rock Bio, Inc group developed RYI-018 (nimacimab), a negative-allosteric modulating antibody targeting CB1R. Early-stage clinical development is ongoing. Clinical trials are assessing safety, tolerability and pharmacokinetics of RYI-018 in patients with non-alcoholic fatty liver disease (phase 1 study, NCT03261739) and diabetic gastroparesis (phase 2a study, NCT03900325). The Goldinch Bio, Inc group also developed GFB-024, another peripherally restricted CB1R inverse agonist monoclonal antibody, targeting patients at high risk of CKD due to severe insulin resistant patients. Phase 1 study in healthy volunteers is ongoing. To date, there is no published studies with RYI-018 nor GFB-024.

## Conclusion

There is now a growing body of evidence for a prominent role of CB1R in a broad range of renal diseases. In metabolic syndrome, obesity and diabetes, CB1R inhibition not only improves metabolic parameters, but also exerted a direct role in the protection of renal function. Since diabetic nephropathy remains one of the main causes of CKD and ESRD and since a large proportion of CKD patients suffer from metabolic syndrome and obesity, the metabolic benefits of CB1R inhibition represents a major advantage in the therapeutic management of these patients. In addition, recent studies highlighted that CB1R also promotes renal fibrosis in non-metabolic nephropathies and that its inhibition reduced the development of renal fibrosis. Rimonabant, belonging to the first generation CB1R antagonists, was approved for clinical use but its development was stopped due to an important risk of severe depression, and it was definitively withdrawn in 2008. To date, second and third generation CB1R antagonists without central nervous side-effects are under development with encouraging experimental data on renal fibrosis prevention. Due to the weak expression of CB1R in healthy kidneys, few side effects of its peripheral blockage are expected, although, as detailed in our review, CB1R could impact GFR and sodium and water balance. There is a growing interest of the industry to develop new CB1R antagonists and some molecules are currently under early-stage clinical phases (phases I and IIa studies), paying a peculiar attention to safety and tolerability. These new pharmacological blockers of CB1R could therefore provide an additional therapeutic toolbox in the management of CKD and renal fibrosis from both metabolic and non-metabolic origin.

## Author Contributions

Both MD and HF authors contributed to the final version of the manuscript. HF supervised the project. All authors contributed to the article and approved the submitted version.

## Conflict of Interest

The authors declare that the research was conducted in the absence of any commercial or financial relationships that could be construed as a potential conflict of interest.

## Abbreviations

2-AG, 2-arachidonoylglycerol; ACC, acetyl-CoA carboxylase; AEA, anandamide; AMPK, AMP-activated kinase; Ang II, angiotensin II; AT1, Ang II type 1 receptors; BBB, blood brain barrier; BP, blood pressure; CAD, chronic kidney allograft dysfunction; CB1R, cannabinoid receptor type 1; CB2R, cannabinoid receptor type 1; CCR2, CC chemokine receptor 2; CKD, chronic kidney disease; COX-2, cyclooxygenase type 2; DAGL, sn-1-specific diacylglycerol lipase; ECS, endocannabinoid system; ESRD, end-stage renal disease; FAAH, fatty acid amide hydrolase; GFR, glomerular filtration rate; GLUT2, glucose transporter 2; HK2, human proximal tubular cell line; HP, hemopressin; HTN, hypertension; IHC, immunohistochemical assay; LKB1, tumor suppressor liver kinase B1; MAGL, monoacylglycerol lipase; MCP-1, monocyte chemoattractant protein-1; NAPE, N-arachidonoyl phosphatidyl ethanolamine; NAPE-PLD, NAPE-specific phospholipase D; PIP2, 2-arachidonoyl-phosphatidylinositol 4,5-bisphosphate; PKA, protein kinase A; PKC, protein kinase C; RAS, renin angiotensin system; SGLT2, sodium glucose co-transporter 2; SHR, spontaneously hypertensive rat; RPTC, renal proximal tubular epithelial cells; RT-PCR, Reverse transcription and polymerase chain reaction; SR141716A, rimonabant; STZ, streptozotocin; TAL, thick ascending limb; THC, tetrahydrocannabinol; TGFβ, transforming growth factor β; TRPV1, transient receptor potential vanilloid type I channels; UUO, unilateral ureteral obstruction; WB, western blot; WIN, WIN55,212-2; ZDF, Zucker diabetic fatty.

## References

[1] “KDIGO Chapter 1: Definition and Classification of CKD”. In: Kidney Int Suppl, vol. 3. p. 19–62. 10.1038/kisup.2012.64 PMC4089693 25018975

[2] Francois H Jacquet A Beaudreuil S Seidowsky A Hebibi H Charpentier B. Emerging Strategies to Preserve Renal Function. J Nephrol (2011) 24:133–41. 10.5301/JN.2011.6355 21319132

[3] Koye DN Shaw JE Reid CM Atkins RC Reutens AT Magliano DJ. Incidence of Chronic Kidney Disease Among People With Diabetes: A Systematic Review of Observational Studies. Diabetes Med J Br Diabetes Assoc (2017) 34:887–901. 10.1111/dme.13324 28164387

[4] Neal B Perkovic V Mahaffey KW de Zeeuw D Fulcher G Erondu N. Canagliflozin and Cardiovascular and Renal Events in Type 2 Diabetes. N Engl J Med (2017) 377:644–57. 10.1056/NEJMoa1611925 28605608

[5] Heerspink HJL Kosiborod M Inzucchi SE Cherney DZI. Renoprotective Effects of Sodium-Glucose Cotransporter-2 Inhibitors. Kidney Int (2018) 94:26–39. 10.1016/j.kint.2017.12.027 29735306

[6] Perkovic V Jardine MJ Neal B Bompoint S Heerspink HJL Charytan DM. Canagliflozin and Renal Outcomes in Type 2 Diabetes and Nephropathy. N Engl J Med (2019) 380:2295–306. 10.1056/NEJMoa1811744 30990260

[7] Toyama T Neuen BL Jun M Ohkuma T Neal B Jardine MJ. Effect of SGLT2 Inhibitors on Cardiovascular, Renal and Safety Outcomes in Patients With Type 2 Diabetes Mellitus and Chronic Kidney Disease: A Systematic Review and Meta-Analysis. Diabetes Obes Metab (2019) 21:1237–50. 10.1111/dom.13648 30697905

[8] Heerspink HJL Stefánsson BV Correa-Rotter R Chertow GM Greene T Hou F-F. Dapagliflozin in Patients With Chronic Kidney Disease. N Engl J Med (2020) 383:1436–46. 10.1056/NEJMoa2024816 32970396

[9] Greco EV Russo G Giandalia A Viazzi F Pontremoli R De Cosmo S. GLP-1 Receptor Agonists and Kidney Protection. Med Kaunas Lith (2019) 55(6):233. 10.3390/medicina55060233 PMC6630923 31159279

[10] Kristensen SL Rørth R Jhund PS Docherty KF Sattar N Preiss D. Cardiovascular, Mortality, and Kidney Outcomes With GLP-1 Receptor Agonists in Patients With Type 2 Diabetes: A Systematic Review and Meta-Analysis of Cardiovascular Outcome Trials. Lancet Diabetes Endocrinol (2019) 7:776–85. 10.1016/S2213-8587(19)30249-9 31422062

[11] Francois H Lecru L. The Role of Cannabinoid Receptors in Renal Diseases. Curr Med Chem (2018) 25:793–801. 10.2174/0929867324666170911170020 28901271

[12] Lecru L Desterke C Grassin-Delyle S Chatziantoniou C Vandermeersch S Devocelle A. Cannabinoid Receptor 1 is a Major Mediator of Renal Fibrosis. Kidney Int (2015) 88:72–84. 10.1038/ki.2015.63 25760323

[13] Barutta F Corbelli A Mastrocola R Gambino R Di Marzo V Pinach S. Cannabinoid Receptor 1 Blockade Ameliorates Albuminuria in Experimental Diabetic Nephropathy. Diabetes (2010) 59:1046–54. 10.2337/db09-1336 PMC2844813 20068137

[14] Nam DH Lee MH Kim JE Song HK Kang YS Lee JE. Blockade of Cannabinoid Receptor 1 Improves Insulin Resistance, Lipid Metabolism, and Diabetic Nephropathy in Db/Db Mice. Endocrinology (2012) 153:1387–96. 10.1210/en.2011-1423 22234468

[15] Jourdan T Szanda G Rosenberg AZ Tam J Earley BJ Godlewski G. Overactive Cannabinoid 1 Receptor in Podocytes Drives Type 2 Diabetic Nephropathy. Proc Natl Acad Sci U S A (2014) 111:E5420–8. 10.1073/pnas.1419901111 PMC4273328 25422468

[16] Udi S Hinden L Earley B Drori A Reuveni N Hadar R. Proximal Tubular Cannabinoid-1 Receptor Regulates Obesity-Induced CKD. J Am Soc Nephrol JASN (2017) 28:3518–32. 10.1681/ASN.2016101085 PMC5698062 28860163

[17] Hinden L Udi S Drori A Gammal A Nemirovski A Hadar R. Modulation of Renal GLUT2 by the Cannabinoid-1 Receptor: Implications for the Treatment of Diabetic Nephropathy. J Am Soc Nephrol JASN (2018) 29:434–48. 10.1681/ASN.2017040371 PMC5791066 29030466

[18] Jourdan T Park JK Varga ZV Pálóczi J Coffey NJ Rosenberg AZ. Cannabinoid-1 Receptor Deletion in Podocytes Mitigates Both Glomerular and Tubular Dysfunction in a Mouse Model of Diabetic Nephropathy. Diabetes Obes Metab (2018) 20:698–708. 10.1111/dom.13150 29106063

[19] Matsuda LA Lolait SJ Brownstein MJ Young AC Bonner TI. Structure of a Cannabinoid Receptor and Functional Expression of the Cloned cDNA. Nature (1990) 346:561–4. 10.1038/346561a0 2165569

[20] Munro S Thomas KL Abu-Shaar M. Molecular Characterization of a Peripheral Receptor for Cannabinoids. Nature (1993) 365:61–5. 10.1038/365061a0 7689702

[21] Ledent C Valverde O Cossu G Petitet F Aubert JF Beslot F. Unresponsiveness to Cannabinoids and Reduced Addictive Effects of Opiates in CB1 Receptor Knockout Mice. Science (1999) 283:401–4. 10.1126/science.283.5400.401 9888857

[22] Howlett AC Blume LC Dalton GD. CB1 Cannabinoid Receptors and Their Associated Proteins. Curr Med Chem (2010) 17:1382. 10.2174/092986710790980023 20166926 PMC3179980

[23] O’Keefe L Simcocks AC Hryciw DH Mathai ML McAinch AJ. The Cannabinoid Receptor 1 and its Role in Influencing Peripheral Metabolism. Diabetes Obes Metab (2014) 16:294–304. 10.1111/dom.12144 23782485

[24] Devane WA Hanus L Breuer A Pertwee RG Stevenson LA Griffin G. Isolation and Structure of a Brain Constituent That Binds to the Cannabinoid Receptor. Science (1992) 258:1946–9. 10.1126/science.1470919 1470919

[25] Mechoulam R Ben-Shabat S Hanus L Ligumsky M Kaminski NE Schatz AR. Identification of an Endogenous 2-Monoglyceride, Present in Canine Gut, That Binds to Cannabinoid Receptors. Biochem Pharmacol (1995) 50:83–90. 10.1016/0006-2952(95)00109-D 7605349

[26] Koura Y Ichihara A Tada Y Kaneshiro Y Okada H Temm CJ. Anandamide Decreases Glomerular Filtration Rate Through Predominant Vasodilation of Efferent Arterioles in Rat Kidneys. J Am Soc Nephrol JASN (2004) 15:1488–94. 10.1097/01.ASN.0000130561.82631.BC 15153559

[27] Larrinaga G Varona A Pérez I Sanz B Ugalde A Cándenas ML. Expression of Cannabinoid Receptors in Human Kidney. Histol Histopathol (2010) 25:1133–8. 10.14670/HH-25.1133 20607655

[28] Dao M Lecru L Vandermeersch S Ferreira M Ferlicot S Posseme K. The Cannabinoid Receptor 1 is Involved in Renal Fibrosis During Chronic Allograft Dysfunction: Proof of Concept. J Cell Mol Med (2019) 23(11):7279–88. 10.1111/jcmm.14570 PMC6815790 31469511

[29] Van Gaal LF Rissanen AM Scheen AJ Ziegler O Rössner S. RIO-Europe Study Group. Effects of the Cannabinoid-1 Receptor Blocker Rimonabant on Weight Reduction and Cardiovascular Risk Factors in Overweight Patients: 1-Year Experience From the RIO-Europe Study. Lancet Lond Engl (2005) 365:1389–97. 10.1016/S0140-6736(05)66374-X 15836887

[30] Després J-P Golay A Sjöström L. Rimonabant in Obesity-Lipids Study Group. Effects of Rimonabant on Metabolic Risk Factors in Overweight Patients With Dyslipidemia. N Engl J Med (2005) 353:2121–34. 10.1056/NEJMoa044537 16291982

[31] Pi-Sunyer FX Aronne LJ Heshmati HM Devin J Rosenstock J. RIO-North America Study Group. Effect of Rimonabant, a Cannabinoid-1 Receptor Blocker, on Weight and Cardiometabolic Risk Factors in Overweight or Obese Patients: RIO-North America: A Randomized Controlled Trial. JAMA (2006) 295:761–75. 10.1001/jama.295.7.761 16478899

[32] Triay J Mundi M Klein S Toledo FG Smith SR Abu-Lebdeh H. Does Rimonabant Independently Affect Free Fatty Acid and Glucose Metabolism? J Clin Endocrinol Metab (2012) 97:819–27. 10.1210/jc.2011-2486 PMC3319222 22170727

[33] Després J-P Ross R Boka G Alméras N Lemieux I. ADAGIO-Lipids Investigators. Effect of Rimonabant on the High-Triglyceride/Low-HDL-Cholesterol Dyslipidemia, Intraabdominal Adiposity, and Liver Fat: The ADAGIO-Lipids Trial. Arterioscler Thromb Vasc Biol (2009) 29:416–23. 10.1161/ATVBAHA.108.176362 19112166

[34] Rosenstock J Hollander P Chevalier S Iranmanesh A. SERENADE Study Group. SERENADE: The Study Evaluating Rimonabant Efficacy in Drug-Naive Diabetic Patients: Effects of Monotherapy With Rimonabant, the First Selective CB1 Receptor Antagonist, on Glycemic Control, Body Weight, and Lipid Profile in Drug-Naive Type 2 Diabetes. Diabetes Care (2008) 31:2169–76. 10.2337/dc08-0386 PMC2571069 18678611

[35] Nissen SE Nicholls SJ Wolski K Rodés-Cabau J Cannon CP Deanfield JE. Effect of Rimonabant on Progression of Atherosclerosis in Patients With Abdominal Obesity and Coronary Artery Disease: The STRADIVARIUS Randomized Controlled Trial. JAMA (2008) 299:1547–60. 10.1001/jama.299.13.1547 18387931

[36] Topol EJ Bousser M-G Fox KAA Creager MA Despres J-P Easton JD. Rimonabant for Prevention of Cardiovascular Events (CRESCENDO): A Randomised, Multicentre, Placebo-Controlled Trial. Lancet Lond Engl (2010) 376:517–23. 10.1016/S0140-6736(10)60935-X 20709233

[37] Cinar R Iyer MR Kunos G. The Therapeutic Potential of Second and Third Generation CB1R Antagonists. Pharmacol Ther (2020) 208:107477. 10.1016/j.pharmthera.2020.107477 31926199 PMC8605822

[38] Tam J Vemuri VK Liu J Bátkai S Mukhopadhyay B Godlewski G. Peripheral CB1 Cannabinoid Receptor Blockade Improves Cardiometabolic Risk in Mouse Models of Obesity. J Clin Invest (2010) 120:2953–66. 10.1172/JCI42551 PMC2912197 20664173

[39] Barutta F Grimaldi S Gambino R Vemuri K Makriyannis A Annaratone L. Dual Therapy Targeting the Endocannabinoid System Prevents Experimental Diabetic Nephropathy. Nephrol Dial Transplant (2017) 32:1655–65. 10.1093/ndt/gfx010 28387811

[40] Barutta F Bellini S Mastrocola R Gambino R Piscitelli F di Marzo V. Reversal of Albuminuria by Combined AM6545 and Perindopril Therapy in Experimental Diabetic Nephropathy. Br J Pharmacol (2018) 175:4371–85. 10.1111/bph.14495 PMC6240130 30184259

[41] Roger C Buch C Muller T Leemput J Demizieux L Passilly-Degrace P. Simultaneous Inhibition of Peripheral CB1R and iNOS Mitigates Obesity-Related Dyslipidemia Through Distinct Mechanisms. Diabetes (2020) 69:2120–32. 10.2337/db20-0078 PMC7506827 32680936

[42] Liu Z Iyer MR Godlewski G Jourdan T Liu J Coffey NJ. Functional Selectivity of a Biased Cannabinoid-1 Receptor (CB1R) Antagonist. ACS Pharmacol Transl Sci (2021) 4(3):1175–87. 10.1021/acsptsci.1c00048 PMC8204328 34151207

[43] Deutsch DG Goligorsky MS Schmid PC Krebsbach RJ Schmid HH Das SK. Production and Physiological Actions of Anandamide in the Vasculature of the Rat Kidney. J Clin Invest (1997) 100:1538–46. 10.1172/JCI119677 PMC508335 9294122

[44] Mukhopadhyay P Pan H Rajesh M Bátkai S Patel V Harvey-White J. CB1 Cannabinoid Receptors Promote Oxidative/Nitrosative Stress, Inflammation and Cell Death in a Murine Nephropathy Model. Br J Pharmacol (2010) 160:657–68. 10.1111/j.1476-5381.2010.00769.x PMC2931565 20590569

[45] Janiak P Poirier B Bidouard JP Cadrouvele C Pierre F Gouraud L. Blockade of Cannabinoid CB1 Receptors Improves Renal Function, Metabolic Profile, and Increased Survival of Obese Zucker Rats. Kidney Int (2007) 72:1345–57. 10.1038/sj.ki.5002540 17882151

[46] Silva GB Atchison DK Juncos LI García NH. Anandamide Inhibits Transport-Related Oxygen Consumption in the Loop of Henle by Activating CB1 Receptors. Am J Physiol Renal Physiol (2013) 304:F376–81. 10.1152/ajprenal.00239.2012 PMC3566492 23220721

[47] Shire D Carillon C Kaghad M Calandra B Rinaldi-Carmona M Le Fur G. An Amino-Terminal Variant of the Central Cannabinoid Receptor Resulting From Alternative Splicing. J Biol Chem (1995) 270:3726–31. 10.1074/jbc.270.8.3726 7876112

[48] Jenkin KA McAinch AJ Grinfeld E Hryciw DH. Role for Cannabinoid Receptors in Human Proximal Tubular Hypertrophy. Cell Physiol Biochem Int J Exp Cell Physiol Biochem Pharmacol (2010) 26:879–86. 10.1159/000323997 21220919

[49] Torlakovic EE Francis G Garratt J Gilks B Hyjek E Ibrahim M. Standardization of Negative Controls in Diagnostic Immunohistochemistry: Recommendations From the International *Ad Hoc* Expert Panel. Appl Immunohistochem Mol Morphol AIMM (2014) 22:241–52. 10.1097/PAI.0000000000000069 PMC4206554 24714041

[50] Torlakovic EE Nielsen S Francis G Garratt J Gilks B Goldsmith JD. Standardization of Positive Controls in Diagnostic Immunohistochemistry: Recommendations From the International Ad Hoc Expert Committee. Appl Immunohistochem Mol Morphol AIMM (2015) 23:1–18. 10.1097/PAI.0000000000000163 25474126

[51] Udi S Hinden L Ahmad M Drori A Iyer MR Cinar R. Dual Inhibition of Cannabinoid CB1 Receptor and Inducible NOS Attenuates Obesity-Induced Chronic Kidney Disease. Br J Pharmacol (2020) 177:110–27. 10.1111/bph.14849 PMC6976880 31454063

[52] Tam J. The Emerging Role of the Endocannabinoid System in the Pathogenesis and Treatment of Kidney Diseases. J Basic Clin Physiol Pharmacol (2016) 27:267–76. 10.1515/jbcpp-2015-0055 26280171

[53] Barutta F Piscitelli F Pinach S Bruno G Gambino R Rastaldi MP. Protective Role of Cannabinoid Receptor Type 2 in a Mouse Model of Diabetic Nephropathy. Diabetes (2011) 60:2386–96. 10.2337/db10-1809 PMC3161308 21810593

[54] Sugiura T Kobayashi Y Oka S Waku K. Biosynthesis and Degradation of Anandamide and 2-Arachidonoylglycerol and Their Possible Physiological Significance. Prostaglandins Leukot Essent Fatty Acids (2002) 66:173–92. 10.1054/plef.2001.0356 12052034

[55] Long JZ LaCava M Jin X Cravatt BF. An Anatomical and Temporal Portrait of Physiological Substrates for Fatty Acid Amide Hydrolase. J Lipid Res (2011) 52:337–44. 10.1194/jlr.M012153 PMC3023554 21097653

[56] Ritter JK Li G Xia M Boini K. Anandamide and its Metabolites: What are Their Roles in the Kidney? Front Biosci Sch Ed (2016) 8:264–77. 10.2741/s461 PMC6267779 27100705

[57] Basith S Cui M Macalino SJY Park J Clavio NAB Kang S. Exploring G Protein-Coupled Receptors (GPCRs) Ligand Space *via* Cheminformatics Approaches: Impact on Rational Drug Design. Front Pharmacol (2018) 9:128. 10.3389/fphar.2018.00128 29593527 PMC5854945

[58] Haspula D Clark MA. Cannabinoid Receptors: An Update on Cell Signaling, Pathophysiological Roles and Therapeutic Opportunities in Neurological, Cardiovascular, and Inflammatory Diseases. Int J Mol Sci (2020) 21(20):7693. 10.3390/ijms21207693 PMC7590033 33080916

[59] Pacher P Bátkai S Kunos G. The Endocannabinoid System as an Emerging Target of Pharmacotherapy. Pharmacol Rev (2006) 58:389–462. 10.1124/pr.58.3.2 16968947 PMC2241751

[60] Natarajan V Schmid PC Reddy PV Zuzarte-Augustin ML Schmid HH. Biosynthesis of N-Acylethanolamine Phospholipids by Dog Brain Preparations. J Neurochem (1983) 41:1303–12. 10.1111/j.1471-4159.1983.tb00825.x 6619867

[61] Di Marzo V Fontana A Cadas H Schinelli S Cimino G Schwartz JC. Formation and Inactivation of Endogenous Cannabinoid Anandamide in Central Neurons. Nature (1994) 372:686–91. 10.1038/372686a0 7990962

[62] Cadas H Gaillet S Beltramo M Venance L Piomelli D. Biosynthesis of an Endogenous Cannabinoid Precursor in Neurons and its Control by Calcium and cAMP. J Neurosci (1996) 16:3934–42. 10.1523/JNEUROSCI.16-12-03934.1996 PMC6578613 8656287

[63] Jin X-H Okamoto Y Morishita J Tsuboi K Tonai T Ueda N. Discovery and Characterization of a Ca2+-Independent Phosphatidylethanolamine N-Acyltransferase Generating the Anandamide Precursor and its Congeners. J Biol Chem (2007) 282:3614–23. 10.1074/jbc.M606369200 17158102

[64] Tsuboi K Uyama T Okamoto Y Ueda N. Endocannabinoids and Related N-Acylethanolamines: Biological Activities and Metabolism. Inflammation Regener (2018) 38:28. 10.1186/s41232-018-0086-5 PMC6166290 30288203

[65] Sugiura T Kondo S Sukagawa A Tonegawa T Nakane S Yamashita A. Enzymatic Synthesis of Anandamide, an Endogenous Cannabinoid Receptor Ligand, Through N-Acylphosphatidylethanolamine Pathway in Testis: Involvement of Ca(2+)-Dependent Transacylase and Phosphodiesterase Activities. Biochem Biophys Res Commun (1996) 218:113–7. 10.1006/bbrc.1996.0020 8573114

[66] Okamoto Y Morishita J Tsuboi K Tonai T Ueda N. Molecular Characterization of a Phospholipase D Generating Anandamide and its Congeners. J Biol Chem (2004) 279:5298–305. 10.1074/jbc.M306642200 14634025

[67] Leung D Saghatelian A Simon GM Cravatt BF. Inactivation of N-Acyl Phosphatidylethanolamine Phospholipase D Reveals Multiple Mechanisms for the Biosynthesis of Endocannabinoids. Biochemistry (2006) 45:4720–6. 10.1021/bi060163l PMC1538545 16605240

[68] Tsuboi K Okamoto Y Ikematsu N Inoue M Shimizu Y Uyama T. Enzymatic Formation of N-Acylethanolamines From N-Acylethanolamine Plasmalogen Through N-Acylphosphatidylethanolamine-Hydrolyzing Phospholipase D-Dependent and -Independent Pathways. Biochim Biophys Acta (2011) 1811:565–77. 10.1016/j.bbalip.2011.07.009 21801852

[69] Leishman E Mackie K Luquet S Bradshaw HB. Lipidomics Profile of a NAPE-PLD KO Mouse Provides Evidence of a Broader Role of This Enzyme in Lipid Metabolism in the Brain. Biochim Biophys Acta (2016) 1861:491–500. 10.1016/j.bbalip.2016.03.003 26956082 PMC4909477

[70] Inoue M Tsuboi K Okamoto Y Hidaka M Uyama T Tsutsumi T. Peripheral Tissue Levels and Molecular Species Compositions of N-Acyl-Phosphatidylethanolamine and its Metabolites in Mice Lacking N-Acyl-Phosphatidylethanolamine-Specific Phospholipase D. J Biochem (Tokyo) (2017) 162:449–58. 10.1093/jb/mvx054 28992041

[71] Simon GM Cravatt BF. Endocannabinoid Biosynthesis Proceeding Through Glycerophospho-N-Acyl Ethanolamine and a Role for Alpha/Beta-Hydrolase 4 in This Pathway. J Biol Chem (2006) 281:26465–72. 10.1074/jbc.M604660200 16818490

[72] Liu J Wang L Harvey-White J Osei-Hyiaman D Razdan R Gong Q. A Biosynthetic Pathway for Anandamide. Proc Natl Acad Sci U S A (2006) 103:13345–50. 10.1073/pnas.0601832103 PMC1557387 16938887

[73] Sun Y-X Tsuboi K Okamoto Y Tonai T Murakami M Kudo I. Biosynthesis of Anandamide and N-Palmitoylethanolamine by Sequential Actions of Phospholipase A2 and Lysophospholipase D. Biochem J (2004) 380:749–56. 10.1042/BJ20040031 PMC1224205 14998370

[74] Sampaio LS Taveira Da Silva R Lima D Sampaio CLC Iannotti FA Mazzarella E. The Endocannabinoid System in Renal Cells: Regulation of Na(+) Transport by CB1 Receptors Through Distinct Cell Signalling Pathways. Br J Pharmacol (2015) 172:4615–25. 10.1111/bph.13050 PMC4594267 25537261

[75] Cravatt BF Giang DK Mayfield SP Boger DL Lerner RA Gilula NB. Molecular Characterization of an Enzyme That Degrades Neuromodulatory Fatty-Acid Amides. Nature (1996) 384:83–7. 10.1038/384083a0 8900284

[76] Arreaza G Devane WA Omeir RL Sajnani G Kunz J Cravatt BF. The Cloned Rat Hydrolytic Enzyme Responsible for the Breakdown of Anandamide Also Catalyzes its Formation *via* the Condensation of Arachidonic Acid and Ethanolamine. Neurosci Lett (1997) 234:59–62. 10.1016/s0304-3940(97)00673-3 9347946

[77] Giang DK Cravatt BF. Molecular Characterization of Human and Mouse Fatty Acid Amide Hydrolases. Proc Natl Acad Sci U S A (1997) 94:2238–42. 10.1073/pnas.94.6.2238 PMC20071 9122178

[78] Wei BQ Mikkelsen TS McKinney MK Lander ES Cravatt BF. A Second Fatty Acid Amide Hydrolase With Variable Distribution Among Placental Mammals. J Biol Chem (2006) 281:36569–78. 10.1074/jbc.M606646200 17015445

[79] Ritter JK Li C Xia M Poklis JL Lichtman AH Abdullah RA. Production and Actions of the Anandamide Metabolite Prostamide E2 in the Renal Medulla. J Pharmacol Exp Ther (2012) 342:770–9. 10.1124/jpet.112.196451 PMC3422528 22685343

[80] Breyer MD Harris RC. Cyclooxygenase 2 and the Kidney. Curr Opin Nephrol Hypertens (2001) 10:89–98. 10.1097/00041552-200101000-00014 11195058

[81] González-Núñez D Solé M Natarajan R Poch E. 12-Lipoxygenase Metabolism in Mouse Distal Convoluted Tubule Cells. Kidney Int (2005) 67:178–86. 10.1111/j.1523-1755.2005.00068.x 15610241

[82] Gohara A Eltaki N Sabry D Murtagh D Jankun J Selman SH. Human 5-, 12- and 15-Lipoxygenase-1 Coexist in Kidney But Show Opposite Trends and Their Balance Changes in Cancer. Oncol Rep (2012) 28:1275–82. 10.3892/or.2012.1924 22825379

[83] Sridar C Snider NT Hollenberg PF. Anandamide Oxidation by Wild-Type and Polymorphically Expressed CYP2B6 and CYP2D6. Drug Metab Dispos Biol Fate Chem (2011) 39:782–8. 10.1124/dmd.110.036707 PMC3082373 21289075

[84] Snider NT Kornilov AM Kent UM Hollenberg PF. Anandamide Metabolism by Human Liver and Kidney Microsomal Cytochrome P450 Enzymes to Form Hydroxyeicosatetraenoic and Epoxyeicosatrienoic Acid Ethanolamides. J Pharmacol Exp Ther (2007) 321:590–7. 10.1124/jpet.107.119321 17272674

[85] Sugiura T Kishimoto S Oka S Gokoh M. Biochemistry, Pharmacology and Physiology of 2-Arachidonoylglycerol, an Endogenous Cannabinoid Receptor Ligand. Prog Lipid Res (2006) 45:405–46. 10.1016/j.plipres.2006.03.003 16678907

[86] Blankman JL Cravatt BF. Chemical Probes of Endocannabinoid Metabolism. Pharmacol Rev (2013) 65:849–71. 10.1124/pr.112.006387 PMC3639726 23512546

[87] Bisogno T Melck D Bobrov M Gretskaya NM Bezuglov VV De Petrocellis L. N-Acyl-Dopamines: Novel Synthetic CB(1) Cannabinoid-Receptor Ligands and Inhibitors of Anandamide Inactivation With Cannabimimetic Activity *In Vitro* and In Vivo. Biochem J (2000) 351 Pt 3:817–24. 10.1042/bj3510817 PMC1221424 11042139

[88] Hanus L Abu-Lafi S Fride E Breuer A Vogel Z Shalev DE. 2-Arachidonyl Glyceryl Ether, an Endogenous Agonist of the Cannabinoid CB1 Receptor. Proc Natl Acad Sci U S A (2001) 98:3662–5. 10.1073/pnas.061029898 PMC31108 11259648

[89] Heimann AS Gomes I Dale CS Pagano RL Gupta A de Souza LL. Hemopressin is an Inverse Agonist of CB1 Cannabinoid Receptors. Proc Natl Acad Sci U S A (2007) 104:20588–93. 10.1073/pnas.0706980105 PMC2154475 18077343

[90] Gomes I Grushko JS Golebiewska U Hoogendoorn S Gupta A Heimann AS. Novel Endogenous Peptide Agonists of Cannabinoid Receptors. FASEB J (2009) 23:3020–9. 10.1096/fj.09-132142 PMC2735371 19380512

[91] Howlett AC Barth F Bonner TI Cabral G Casellas P Devane WA. International Union of Pharmacology. XXVII. Classification of Cannabinoid Receptors. Pharmacol Rev (2002) 54:161–202. 10.1124/pr.54.2.161 12037135

[92] Zygmunt PM Petersson J Andersson DA Chuang H Sørgård M Di Marzo V. Vanilloid Receptors on Sensory Nerves Mediate the Vasodilator Action of Anandamide. Nature (1999) 400:452–7. 10.1038/22761 10440374

[93] Pegorini S Zani A Braida D Guerini-Rocco C Sala M. Vanilloid VR1 Receptor is Involved in Rimonabant-Induced Neuroprotection. Br J Pharmacol (2006) 147:552–9. 10.1038/sj.bjp.0706656 PMC1616983 16444289

[94] Hansen HH Azcoitia I Pons S Romero J García-Segura LM Ramos JA. Blockade of Cannabinoid CB(1) Receptor Function Protects Against *In Vivo* Disseminating Brain Damage Following NMDA-Induced Excitotoxicity. J Neurochem (2002) 82:154–8. 10.1046/j.1471-4159.2002.00961.x 12091476

[95] De Petrocellis L Bisogno T Maccarrone M Davis JB Finazzi-Agro A Di Marzo V. The Activity of Anandamide at Vanilloid VR1 Receptors Requires Facilitated Transport Across the Cell Membrane and is Limited by Intracellular Metabolism. J Biol Chem (2001) 276:12856–63. 10.1074/jbc.M008555200 11278420

[96] Köfalvi A Vizi ES Ledent C Sperlágh B. Cannabinoids Inhibit the Release of [3H]Glutamate From Rodent Hippocampal Synaptosomes *via* a Novel CB1 Receptor-Independent Action. Eur J Neurosci (2003) 18:1973–8. 10.1046/j.1460-9568.2003.02897.x 14622229

[97] Berger C Schmid PC Schabitz W-R Wolf M Schwab S Schmid HHO. Massive Accumulation of N-Acylethanolamines After Stroke. Cell Signalling in Acute Cerebral Ischemia? J Neurochem (2004) 88:1159–67. 10.1046/j.1471-4159.2003.02244.x 15009671

[98] Berdyshev EV Schmid PC Krebsbach RJ Hillard CJ Huang C Chen N. Cannabinoid-Receptor-Independent Cell Signalling by N-Acylethanolamines. Biochem J (2001) 360:67–75. 10.1042/0264-6021:3600067 11695993 PMC1222203

[99] Hermann H De Petrocellis L Bisogno T Schiano Moriello A Lutz B Di Marzo V. Dual Effect of Cannabinoid CB1 Receptor Stimulation on a Vanilloid VR1 Receptor-Mediated Response. Cell Mol Life Sci CMLS (2003) 60:607–16. 10.1007/s000180300052 PMC11138629 12737320

[100] Chemin J Monteil A Perez-Reyes E Nargeot J Lory P. Direct Inhibition of T-Type Calcium Channels by the Endogenous Cannabinoid Anandamide. EMBO J (2001) 20:7033–40. 10.1093/emboj/20.24.7033 PMC125779 11742980

[101] Markó L Mannaa M Haschler TN Krämer S Gollasch M. Renoprotection: Focus on TRPV1, TRPV4, TRPC6 and TRPM2. Acta Physiol Oxf Engl (2017) 219:589–612. 10.1111/apha.12828 28028935

[102] Chen L Markó L Kaßmann M Zhu Y Wu K Gollasch M. Role of TRPV1 Channels in Ischemia/Reperfusion-Induced Acute Kidney Injury. PLoS One (2014) 9:e109842. 10.1371/journal.pone.0109842 25330307 PMC4201466

[103] Ueda K Tsuji F Hirata T Takaoka M Matsumura Y. Preventive Effect of TRPV1 Agonists Capsaicin and Resiniferatoxin on Ischemia/Reperfusion-Induced Renal Injury in Rats. J Cardiovasc Pharmacol (2008) 51:513–20. 10.1097/FJC.0b013e31816f6884 18460982

[104] Tsagogiorgas C Wedel J Hottenrott M Schneider MO Binzen U Greffrath W. N-Octanoyl-Dopamine is an Agonist at the Capsaicin Receptor TRPV1 and Mitigates Ischemia-Induced [Corrected] Acute Kidney Injury in Rat. PLoS One (2012) 7:e43525. 10.1371/journal.pone.0043525 22916273 PMC3423369

[105] Cinar R Iyer MR Liu Z Cao Z Jourdan T Erdelyi K. Hybrid Inhibitor of Peripheral Cannabinoid-1 Receptors and Inducible Nitric Oxide Synthase Mitigates Liver Fibrosis. JCI Insight (2016) 1(11):e87336. 10.1172/jci.insight.87336 PMC4979564 27525312

[106] Showalter VM Compton DR Martin BR Abood ME. Evaluation of Binding in a Transfected Cell Line Expressing a Peripheral Cannabinoid Receptor (CB2): Identification of Cannabinoid Receptor Subtype Selective Ligands. J Pharmacol Exp Ther (1996) 278:989–99. 8819477

[107] Pertwee RG Howlett AC Abood ME Alexander SPH Marzo VD Elphick MR. International Union of Basic and Clinical Pharmacology. LXXIX. Cannabinoid Receptors and Their Ligands: Beyond CB1 and CB2. Pharmacol Rev (2010) 62:588–631. 10.1124/pr.110.003004 21079038 PMC2993256

[108] Rinaldi-Carmona M Barth F Héaulme M Shire D Calandra B Congy C. SR141716A, a Potent and Selective Antagonist of the Brain Cannabinoid Receptor. FEBS Lett (1994) 350:240–4. 10.1016/0014-5793(94)00773-x 8070571

[109] Chorvat RJ Berbaum J Seriacki K McElroy JF. JD-5006 and JD-5037: Peripherally Restricted (PR) Cannabinoid-1 Receptor Blockers Related to SLV-319 (Ibipinabant) as Metabolic Disorder Therapeutics Devoid of CNS Liabilities. Bioorg Med Chem Lett (2012) 22:6173–80. 10.1016/j.bmcl.2012.08.004 22959249

[110] Tam J Cinar R Liu J Godlewski G Wesley D Jourdan T. Peripheral Cannabinoid-1 Receptor Inverse Agonism Reduces Obesity by Reversing Leptin Resistance. Cell Metab (2012) 16:167–79. 10.1016/j.cmet.2012.07.002 PMC3832894 22841573

[111] Cluny NL Vemuri VK Chambers AP Limebeer CL Bedard H Wood JT. A Novel Peripherally Restricted Cannabinoid Receptor Antagonist, AM6545, Reduces Food Intake and Body Weight, But Does Not Cause Malaise, in Rodents. Br J Pharmacol (2010) 161:629–42. 10.1111/j.1476-5381.2010.00908.x PMC2990160 20880401

[112] Gatley SJ Gifford AN Volkow ND Lan R Makriyannis A. 123I-Labeled AM251: A Radioiodinated Ligand Which Binds *in vivo* to mouse brain cannabinoid CB1 receptors. Eur J Pharmacol (1996) 307:331–8. 10.1016/0014-2999(96)00279-8 8836622

[113] Lan R Liu Q Fan P Lin S Fernando SR McCallion D. Structure-Activity Relationships of Pyrazole Derivatives as Cannabinoid Receptor Antagonists. J Med Chem (1999) 42:769–76. 10.1021/jm980363y 10052983

[114] Lange JHM Coolen HKAC van Stuivenberg HH Dijksman JAR Herremans AHJ Ronken E. Synthesis, Biological Properties, and Molecular Modeling Investigations of Novel 3,4-Diarylpyrazolines as Potent and Selective CB(1) Cannabinoid Receptor Antagonists. J Med Chem (2004) 47:627–43. 10.1021/jm031019q 14736243

[115] Lan R Gatley J Lu Q Fan P Fernando SR Volkow ND. Design and Synthesis of the CB1 Selective Cannabinoid Antagonist AM281: A Potential Human SPECT Ligand. AAPS PharmSci (1999) 1:E4. 10.1208/ps010204 11741201 PMC2761119

[116] Abadji V Lin S Taha G Griffin G Stevenson LA Pertwee RG. (R)-Methanandamide: A Chiral Novel Anandamide Possessing Higher Potency and Metabolic Stability. J Med Chem (1994) 37:1889–93. 10.1021/jm00038a020 8021930

[117] Rinaldi-Carmona M Barth F Millan J Derocq JM Casellas P Congy C. SR 144528, the First Potent and Selective Antagonist of the CB2 Cannabinoid Receptor. J Pharmacol Exp Ther (1998) 284:644–50. 9454810

[118] Yao BB Mukherjee S Fan Y Garrison TR Daza AV Grayson GK. In Vitro Pharmacological Characterization of AM1241: A Protean Agonist at the Cannabinoid CB2 Receptor? Br J Pharmacol (2006) 149:145–54. 10.1038/sj.bjp.0706838 PMC2013801 16894349

[119] Huffman JW Liddle J Yu S Aung MM Abood ME Wiley JL. 3-(1’,1’-Dimethylbutyl)-1-Deoxy-Delta8-THC and Related Compounds: Synthesis of Selective Ligands for the CB2 Receptor. Bioorg Med Chem (1999) 7:2905–14. 10.1016/s0968-0896(99)00219-9 10658595

[120] Hosohata Y Quock RM Hosohata K Makriyannis A Consroe P Roeske WR. AM630 Antagonism of Cannabinoid-Stimulated [35S]GTP Gamma S Binding in the Mouse Brain. Eur J Pharmacol (1997) 321:R1–3. 10.1016/s0014-2999(97)00047-2 9083796

[121] Ames F. A Clinical and Metabolic Study of Acute Intoxication With Cannabis Sativa and its Role in the Model Psychoses. J Ment Sci (1958) 104:972–99. 10.1192/bjp.104.437.972 13621144

[122] Sofia RD Knobloch LC Harakal JJ Erikson DJ. Comparative Diuretic Activity of Delta9-Tetrahydrocannabinol, Cannabidiol, Cannabinol and Hydrochlorothiazide in the Rat. Arch Int Pharmacodyn Ther (1977) 225:77–87. 849066

[123] Kapusta DR Obih JC. Central Kappa Opioid Receptor-Evoked Changes in Renal Function in Conscious Rats: Participation of Renal Nerves. J Pharmacol Exp Ther (1993) 267:197–204. 8229746

[124] Kapusta DR Obih JC. Central Kappa Opioids Blunt the Renal Excretory Responses to Volume Expansion by a Renal Nerve-Dependent Mechanism. J Pharmacol Exp Ther (1995) 273:199–205. 7714767

[125] Di S Boudaba C Popescu IR Weng F-J Harris C Marcheselli VL. Activity-Dependent Release and Actions of Endocannabinoids in the Rat Hypothalamic Supraoptic Nucleus. J Physiol (2005) 569:751–60. 10.1113/jphysiol.2005.097477 PMC1464259 16239276

[126] Soya A Serino R Fujihara H Onaka T Ozaki Y Saito T. Cannabinoids Modulate Synaptic Activity in the Rat Supraoptic Nucleus. J Neuroendocrinol (2005) 17:609–15. 10.1111/j.1365-2826.2005.01350.x 16101900

[127] Chopda GR Vemuri VK Sharma R Thakur GA Makriyannis A Paronis CA. Diuretic Effects of Cannabinoid Agonists in Mice. Eur J Pharmacol (2013) 721:64–9. 10.1016/j.ejphar.2013.09.053 PMC3872476 24099963

[128] Li J Wang DH. Differential Mechanisms Mediating Depressor and Diuretic Effects of Anandamide. J Hypertens (2006) 24:2271–6. 10.1097/01.hjh.0000249706.42230.a8 17053550

[129] Paronis CA Thakur GA Bajaj S Nikas SP Vemuri VK Makriyannis A. Diuretic Effects of Cannabinoids. J Pharmacol Exp Ther (2013) 344:8–14. 10.1124/jpet.112.199331 23019138 PMC3533417

[130] Ritter JK Ahmad A Mummalaneni S Daneva Z Dempsey SK Li N. Mechanism of Diuresis and Natriuresis by Cannabinoids: Evidence for Inhibition of Na+-K+-ATPase in Mouse Kidney Thick Ascending Limb Tubules. J Pharmacol Exp Ther (2021) 376:1–11. 10.1124/jpet.120.000163 33087396 PMC7745087

[131] Sampaio LS Iannotti FA Veneziani L Borelli-Tôrres RT De Maio F Piscitelli F. Experimental Ischemia/Reperfusion Model Impairs Endocannabinoid Signaling and Na+/K+ ATPase Expression and Activity in Kidney Proximal Tubule Cells. Biochem Pharmacol (2018) 154:482–91. 10.1016/j.bcp.2018.06.005 29890144

[132] Vollmer RR Cavero I Ertel RJ Solomon TA Buckley JP. Role of the Central Autonomic Nervous System in the Hypotension and Bradycardia Induced by (-)-Delta 9-Trans-Tetrahydrocannabinol. J Pharm Pharmacol (1974) 26:186–92. 10.1111/j.2042-7158.1974.tb09252.x 4151077

[133] Lake KD Compton DR Varga K Martin BR Kunos G. Cannabinoid-Induced Hypotension and Bradycardia in Rats Mediated by CB1-Like Cannabinoid Receptors. J Pharmacol Exp Ther (1997) 281:1030–7. 9190833

[134] Benowitz NL Jones RT. Cardiovascular Effects of Prolonged Delta-9-Tetrahydrocannabinol Ingestion. Clin Pharmacol Ther (1975) 18:287–97. 10.1002/cpt1975183287 1164818

[135] Cavero I Buckley JP Jandhyala BS. Hemodynamic and Myocardial Effects of (-)-Delta9-Trans-Tetrahydrocannabinol in Anesthetized Dogs. Eur J Pharmacol (1973) 24:243–51. 10.1016/0014-2999(73)90078-2 4765747

[136] Varga K Lake K Martin BR Kunos G. Novel Antagonist Implicates the CB1 Cannabinoid Receptor in the Hypotensive Action of Anandamide. Eur J Pharmacol (1995) 278:279–83. 10.1016/0014-2999(95)00181-j 7589169

[137] Siqueira SW Lapa AJ Ribeiro do Valle J. The Triple Effect Induced by Delta 9-Tetrahydrocannabinol on the Rat Blood Pressure. Eur J Pharmacol (1979) 58:351–7. 10.1016/0014-2999(79)90305-4 510372

[138] Malinowska B Baranowska-Kuczko M Schlicker E. Triphasic Blood Pressure Responses to Cannabinoids: Do We Understand the Mechanism? Br J Pharmacol (2012) 165:2073–88. 10.1111/j.1476-5381.2011.01747.x PMC3413845 22022923

[139] Vidrio H Sánchez-Salvatori MA Medina M. Cardiovascular Effects of (-)-11-OH-Delta 8-Tetrahydrocannabinol-Dimethylheptyl in Rats. J Cardiovasc Pharmacol (1996) 28:332–6. 10.1097/00005344-199608000-00022 8856492

[140] Seagard JL Dean C Patel S Rademacher DJ Hopp FA Schmeling WT. Anandamide Content and Interaction of Endocannabinoid/GABA Modulatory Effects in the NTS on Baroreflex-Evoked Sympathoinhibition. Am J Physiol Heart Circ Physiol (2004) 286:H992–1000. 10.1152/ajpheart.00870.2003 14615281

[141] Gyombolai P Pap D Turu G Catt KJ Bagdy G Hunyady L. Regulation of Endocannabinoid Release by G Proteins: A Paracrine Mechanism of G Protein-Coupled Receptor Action. Mol Cell Endocrinol (2012) 353:29–36. 10.1016/j.mce.2011.10.011 22075205 PMC4169275

[142] Bonz A Laser M Küllmer S Kniesch S Babin-Ebell J Popp V. Cannabinoids Acting on CB1 Receptors Decrease Contractile Performance in Human Atrial Muscle. J Cardiovasc Pharmacol (2003) 41:657–64. 10.1097/00005344-200304000-00020 12658069

[143] White R Ho WS Bottrill FE Ford WR Hiley CR. Mechanisms of Anandamide-Induced Vasorelaxation in Rat Isolated Coronary Arteries. Br J Pharmacol (2001) 134:921–9. 10.1038/sj.bjp.0704333 PMC1573021 11606334

[144] O’Sullivan SE Kendall DA Randall MD. The Effects of Delta9-Tetrahydrocannabinol in Rat Mesenteric Vasculature, and its Interactions With the Endocannabinoid Anandamide. Br J Pharmacol (2005) 145:514–26. 10.1038/sj.bjp.0706218 PMC1576168 15821751

[145] O’Sullivan SE Kendall DA Randall MD. Vascular Effects of Delta 9-Tetrahydrocannabinol (THC), Anandamide and N-Arachidonoyldopamine (NADA) in the Rat Isolated Aorta. Eur J Pharmacol (2005) 507:211–21. 10.1016/j.ejphar.2004.11.056 15659311

[146] Wagner JA Abesser M Karcher J Laser M Kunos G. Coronary Vasodilator Effects of Endogenous Cannabinoids in Vasopressin-Preconstricted Unpaced Rat Isolated Hearts. J Cardiovasc Pharmacol (2005) 46:348–55. 10.1097/01.fjc.0000175437.87283.f2 16116341

[147] Dannert MT Alsasua A Herradon E Martín MI López-Miranda V. Vasorelaxant Effect of Win 55,212-2 in Rat Aorta: New Mechanisms Involved. Vascul Pharmacol (2007) 46:16–23. 10.1016/j.vph.2006.06.005 16860612

[148] Szekeres M Nádasy GL Soltész-Katona E Hunyady L. Control of Myogenic Tone and Agonist Induced Contraction of Intramural Coronary Resistance Arterioles by Cannabinoid Type 1 Receptors and Endocannabinoids. Prostaglandins Other Lipid Mediat (2018) 134:77–83. 10.1016/j.prostaglandins.2017.10.001 29031792

[149] Járai Z Wagner JA Goparaju SK Wang L Razdan RK Sugiura T. Cardiovascular Effects of 2-Arachidonoyl Glycerol in Anesthetized Mice. Hypertens Dallas Tex 1979 (2000) 35:679–84. 10.1161/01.hyp.35.2.679 10679517

[150] Calignano A La Rana G Beltramo M Makriyannis A Piomelli D. Potentiation of Anandamide Hypotension by the Transport Inhibitor, AM404. Eur J Pharmacol (1997) 337:R1–2. 10.1016/s0014-2999(97)01297-1 9389389

[151] Bátkai S Pacher P Osei-Hyiaman D Radaeva S Liu J Harvey-White J. Endocannabinoids Acting at Cannabinoid-1 Receptors Regulate Cardiovascular Function in Hypertension. Circulation (2004) 110:1996–2002. 10.1161/01.CIR.0000143230.23252.D2 15451779 PMC2756479

[152] Kosersky DS. Antihypertensive Effects of Delta9-Tetrahydrocannabinol. Arch Int Pharmacodyn Ther (1978) 233:76–81. 686909

[153] Lake KD Martin BR Kunos G Varga K. Cardiovascular Effects of Anandamide in Anesthetized and Conscious Normotensive and Hypertensive Rats. Hypertens Dallas Tex 1979 (1997) 29:1204–10. 10.1161/01.hyp.29.5.1204 9149688

[154] Szekeres M Nádasy GL Turu G Soltész-Katona E Tóth ZE Balla A. Angiotensin II Induces Vascular Endocannabinoid Release, Which Attenuates its Vasoconstrictor Effect *via* CB1 cannabinoid receptors. J Biol Chem (2012) 287:31540–50. 10.1074/jbc.M112.346296 PMC3438986 22787147

[155] Szekeres M Nádasy GL Turu G Soltész-Katona E Benyó Z Offermanns S. Endocannabinoid-Mediated Modulation of Gq/11 Protein-Coupled Receptor Signaling-Induced Vasoconstriction and Hypertension. Mol Cell Endocrinol (2015) 403:46–56. 10.1016/j.mce.2015.01.012 25595485

[156] Schrankl J Fuchs M Broeker K Daniel C Kurtz A Wagner C. Localization of Angiotensin II Type 1 Receptor Gene Expression in Rodent and Human Kidneys. Am J Physiol Renal Physiol (2021) 320:F644–53. 10.1152/ajprenal.00550.2020 33615887

[157] Crowley SD Coffman TM. In Hypertension, the Kidney Breaks Your Heart. Curr Cardiol Rep (2008) 10:470–6. 10.1007/s11886-008-0074-5 18950556

[158] Rozenfeld R Gupta A Gagnidze K Lim MP Gomes I Lee-Ramos D. AT1R-Cb₁R Heteromerization Reveals a New Mechanism for the Pathogenic Properties of Angiotensin II. EMBO J (2011) 30:2350–63. 10.1038/emboj.2011.139 PMC3116274 21540834

[159] Teixeira-Clerc F Julien B Grenard P Tran Van Nhieu J Deveaux V Li L. CB1 Cannabinoid Receptor Antagonism: A New Strategy for the Treatment of Liver Fibrosis. Nat Med (2006) 12:671–6. 10.1038/nm1421 16715087

[160] Patsenker E Stoll M Millonig G Agaimy A Wissniowski T Schneider V. Cannabinoid Receptor Type I Modulates Alcohol-Induced Liver Fibrosis. Mol Med Camb Mass (2011) 17:1285–94. 10.2119/molmed.2011.00149 PMC3321809 21863215

[161] Dai E Zhang J Zhang D Yang L Wang Y Jiang X. Rimonabant Inhibits Proliferation, Collagen Secretion and Induces Apoptosis in Hepatic Stellate Cells. Hepatogastroenterology (2014) 61:2052–61. 25713910

[162] Slavic S Lauer D Sommerfeld M Kemnitz UR Grzesiak A Trappiel M. Cannabinoid Receptor 1 Inhibition Improves Cardiac Function and Remodelling After Myocardial Infarction and in Experimental Metabolic Syndrome. J Mol Med Berl Ger (2013) 91:811–23. 10.1007/s00109-013-1034-0 23636507

[163] Bronova I Smith B Aydogan B Weichselbaum RR Vemuri K Erdelyi K. Protection From Radiation-Induced Pulmonary Fibrosis by Peripheral Targeting of Cannabinoid Receptor-1. Am J Respir Cell Mol Biol (2015) 53:555–62. 10.1165/rcmb.2014-0331OC PMC4742897 26426981

[164] Cinar R Gochuico BR Iyer MR Jourdan T Yokoyama T Park JK. Cannabinoid CB1 Receptor Overactivity Contributes to the Pathogenesis of Idiopathic Pulmonary Fibrosis. JCI Insight (2017) 2(8):e92281. 10.1172/jci.insight.92281 PMC5396529 28422760

[165] Lazzerini PE Natale M Gianchecchi E Capecchi PL Montilli C Zimbone S. Adenosine A2A Receptor Activation Stimulates Collagen Production in Sclerodermic Dermal Fibroblasts Either Directly and Through a Cross-Talk With the Cannabinoid System. J Mol Med Berl Ger (2012) 90:331–42. 10.1007/s00109-011-0824-5 22033526

[166] Julien B Grenard P Teixeira-Clerc F Van Nhieu JT Li L Karsak M. Antifibrogenic Role of the Cannabinoid Receptor CB2 in the Liver. Gastroenterology (2005) 128:742–55. 10.1053/j.gastro.2004.12.050 15765409

[167] Defer N Wan J Souktani R Escoubet B Perier M Caramelle P. The Cannabinoid Receptor Type 2 Promotes Cardiac Myocyte and Fibroblast Survival and Protects Against Ischemia/Reperfusion-Induced Cardiomyopathy. FASEB J (2009) 23:2120–30. 10.1096/fj.09-129478 19246487

[168] Servettaz A Kavian N Nicco C Deveaux V Chéreau C Wang A. Targeting the Cannabinoid Pathway Limits the Development of Fibrosis and Autoimmunity in a Mouse Model of Systemic Sclerosis. Am J Pathol (2010) 177:187–96. 10.2353/ajpath.2010.090763 PMC2893662 20508030

[169] Zhou L Zhou S Yang P Tian Y Feng Z Xie X-Q. Targeted Inhibition of the Type 2 Cannabinoid Receptor is a Novel Approach to Reduce Renal Fibrosis. Kidney Int (2018) 94:756–72. 10.1016/j.kint.2018.05.023 PMC6151282 30093080

[170] Zhou S Wu Q Lin X Ling X Miao J Liu X. Cannabinoid Receptor Type 2 Promotes Kidney Fibrosis Through Orchestrating β-Catenin Signaling. Kidney Int (2021) 99:364–81. 10.1016/j.kint.2020.09.025 33152447

[171] Barutta F Grimaldi S Franco I Bellini S Gambino R Pinach S. Deficiency of Cannabinoid Receptor of Type 2 Worsens Renal Functional and Structural Abnormalities in Streptozotocin-Induced Diabetic Mice. Kidney Int (2014) 86(5):979–90. 10.1038/ki.2014.165 24827776

[172] Chin E Zhou J Bondy C. Anatomical and Developmental Patterns of Facilitative Glucose Transporter Gene Expression in the Rat Kidney. J Clin Invest (1993) 91:1810–5. 10.1172/JCI116392 PMC288162 8473519

[173] Thorens B. Glucose Transporters in the Regulation of Intestinal, Renal, and Liver Glucose Fluxes. Am J Physiol (1996) 270:G541–553. 10.1152/ajpgi.1996.270.4.G541 8928783

[174] Marks J Carvou NJC Debnam ES Srai SK Unwin RJ. Diabetes Increases Facilitative Glucose Uptake and GLUT2 Expression at the Rat Proximal Tubule Brush Border Membrane. J Physiol (2003) 553:137–45. 10.1113/jphysiol.2003.046268 PMC2343472 12963802

[175] Chichger H Cleasby ME Srai SK Unwin RJ Debnam ES Marks J. Experimental Type II Diabetes and Related Models of Impaired Glucose Metabolism Differentially Regulate Glucose Transporters at the Proximal Tubule Brush Border Membrane. Exp Physiol (2016) 101:731–42. 10.1113/EP085670 27164183

[176] Rahmoune H Thompson PW Ward JM Smith CD Hong G Brown J. Glucose Transporters in Human Renal Proximal Tubular Cells Isolated From the Urine of Patients With non-Insulin-Dependent Diabetes. Diabetes (2005) 54:3427–34. 10.2337/diabetes.54.12.3427 16306358

[177] Chin E Zamah AM Landau D Grønbcek H Flyvbjerg A LeRoith D. Changes in Facilitative Glucose Transporter Messenger Ribonucleic Acid Levels in the Diabetic Rat Kidney. Endocrinology (1997) 138:1267–75. 10.1210/endo.138.3.5015 9048635

[178] Cohen M Kitsberg D Tsytkin S Shulman M Aroeti B Nahmias Y. Live Imaging of GLUT2 Glucose-Dependent Trafficking and its Inhibition in Polarized Epithelial Cysts. Open Biol (2014) 4(7):140091. 10.1098/rsob.140091 25056286 PMC4118605

[179] Haas M. Chronic Allograft Nephropathy or Interstitial Fibrosis and Tubular Atrophy: What is in a Name? Curr Opin Nephrol Hypertens (2014) 23:245–50. 10.1097/01.mnh.0000444811.26884.2d 24626060

[180] Racusen LC Regele H. The Pathology of Chronic Allograft Dysfunction. Kidney Int Suppl (2010) (119):S27–32. 10.1038/ki.2010.419 21116314

[181] Pascual J Pérez-Sáez MJ Mir M Crespo M. Chronic Renal Allograft Injury: Early Detection, Accurate Diagnosis and Management. Transplant Rev Orlando Fla (2012) 26:280–90. 10.1016/j.trre.2012.07.002 22902496

[182] Heemann U Lutz J. Pathophysiology and Treatment Options of Chronic Renal Allograft Damage. Nephrol Dial Transplant (2013) 28:2438–46. 10.1093/ndt/gft087 23625970

[183] Maluf DG Dumur CI Suh JL Lee JK Cathro HP King AL. Evaluation of Molecular Profiles in Calcineurin Inhibitor Toxicity Post-Kidney Transplant: Input to Chronic Allograft Dysfunction. Am J Transplant (2014) 14:1152–63. 10.1111/ajt.12696 PMC4377109 24698514

[184] Venner JM Famulski KS Reeve J Chang J Halloran PF. Relationships Among Injury, Fibrosis, and Time in Human Kidney Transplants. JCI Insight (2016) 1:e85323. 10.1172/jci.insight.85323 27699214 PMC5033890

[185] El-Zoghby ZM Stegall MD Lager DJ Kremers WK Amer H Gloor JM. Identifying Specific Causes of Kidney Allograft Loss. Am J Transplant (2009) 9:527–35. 10.1111/j.1600-6143.2008.02519.x 19191769

[186] Sellarés J de Freitas DG Mengel M Reeve J Einecke G Sis B. Understanding the Causes of Kidney Transplant Failure: The Dominant Role of Antibody-Mediated Rejection and Nonadherence. Am J Transplant Surg (2012) 12:388–99. 10.1111/j.1600-6143.2011.03840.x 22081892

[187] Roufosse C Simmonds N Groningen MC Haas M Henriksen KJ Horsfield C. A 2018 Reference Guide to the Banff Classification of Renal Allograft Pathology. Transplantation (2018) 102(11):1795–814. 10.1097/TP.0000000000002366 PMC7597974 30028786

[188] Hirsch S Tam J. Cannabis: From a Plant That Modulates Feeding Behaviors Toward Developing Selective Inhibitors of the Peripheral Endocannabinoid System for the Treatment of Obesity and Metabolic Syndrome. Toxins (2019) 11:E275. 10.3390/toxins11050275 31096702 PMC6563239

[189] Chorvat RJ. Peripherally Restricted CB1 Receptor Blockers. Bioorg Med Chem Lett (2013) 23:4751–60. 10.1016/j.bmcl.2013.06.066 23902803

[190] Iyer MR Cinar R Katz A Gao M Erdelyi K Jourdan T. Design, Synthesis, and Biological Evaluation of Novel, Non-Brain-Penetrant, Hybrid Cannabinoid CB1R Inverse Agonist/Inducible Nitric Oxide Synthase (iNOS) Inhibitors for the Treatment of Liver Fibrosis. J Med Chem (2017) 60:1126–41. 10.1021/acs.jmedchem.6b01504 28085283

[191] Martin B Caron N Jadot I Colombaro V Federici G Depommier C. Evaluation of Inducible Nitric Oxide Synthase Inhibition on Kidney Function and Structure in High-Fat Diet-Induced Kidney Disease. Exp Physiol (2018) 103:125–40. 10.1113/EP086594 28944982

[192] Ozbek E Ilbey YO Ozbek M Simsek A Cekmen M Somay A. Melatonin Attenuates Unilateral Ureteral Obstruction-Induced Renal Injury by Reducing Oxidative Stress, iNOS, MAPK, and NF-kB Expression. J Endourol (2009) 23:1165–73. 10.1089/end.2009.0035 19530942

